# Efficacy of praziquantel has been maintained over four decades (from 1977 to 2018): A systematic review and meta-analysis of factors influence its efficacy

**DOI:** 10.1371/journal.pntd.0009189

**Published:** 2021-03-17

**Authors:** Mizuho Fukushige, Margo Chase-Topping, Mark E. J. Woolhouse, Francisca Mutapi

**Affiliations:** 1 Institute of Immunology and Infection Research, Centre for Immunity, Infection & Evolution, Edinburgh Medical School: Biomedical Sciences, University of Edinburgh, Edinburgh, United Kingdom; 2 Faculty of Medicine, University of Tsukuba, Ibaraki, Japan; 3 Roslin Institute and Royal (Dick) School of Veterinary Studies, University of Edinburgh, Edinburgh, United Kingdom; 4 Usher Institute, University of Edinburgh, Edinburgh, United Kingdom; 5 Institute of Immunology and Infection Research, Centre for Immunity, Infection & Evolution, School of Biological Sciences, University of Edinburgh, Edinburgh, United Kingdom; 6 NIHR Global Health Research Unit Tackling Infections to Benefit Africa (TIBA), University of Edinburgh, Edinburgh, United Kingdom; University of Pennsylvania, UNITED STATES

## Abstract

**Background:**

The antihelminthic drug praziquantel has been used as the drug of choice for treating schistosome infection for more than 40 years. Although some epidemiological studies have reported low praziquantel efficacy in cure rate (CR) and/or egg reduction rate (ERR), there is no consistent robust evidence of the development of schistosome resistance to praziquantel (PZQ). There is need to determine factors that lead to variable treatment CR and/or ERR. Therefore, we conducted a systematic review and meta-analysis to review CR and ERR as well as identify their predictors.

**Methodology/Principal findings:**

In this systematic review and meta-analysis, a literature review was conducted using Biosis Citation Index, Data Citation Index, MEDLINE, and Web of Science Core Collection all of which were provided through Web of Science. Alongside these, EMBASE, and CAB abstracts were searched to identify relevant articles. Random effect meta-regression models were used to identify the factors that influence CR and/or ERR by considering differences in host characteristics and drug dose. In total, 12,127 potential articles were screened and 146 eligible articles (published from 1979 to 2020) were identified and included for the meta-analysis. We found that there has been no significant reduction in CR or ERR over the study period. The results showed more variability in CR, compared with ERR which was more consistent and remained high. The results showed a positive effect of “PZQ treatment dose” with the current recommended dose of 40 mg/kg body weight achieving 57% to 88% CR depending on schistosome species, age of participants, and number of parasitological samples used for diagnosis, and ERR of 95%.

**Conclusions/Significance:**

Based on a review of over 40 years of research there is no evidence to support concerns about schistosomes developing resistance to PZQ. These results indicate that PZQ remains effective in treating schistosomiasis.

## Author summary

Schistosomiasis is a water-borne disease caused by a group of flatworm parasites known as schistosomes. The drug praziquantel has been used since the 1970s to treat schistosomiasis with some variable measures of efficacy reported. Reasons for the variation can be related to host attributes or parasite attributes. We conducted this study to determine factors affecting drug efficacy and if the efficacy has altered in the past four decades.

We identified 146 scientific articles that reported efficacy levels of praziquantel treatment for schistosomiasis as determined by cure rate (CR) and/or egg reduction rate (ERR). We conclude that praziquantel treatment is still effective for schistosomiasis even after four decades of usage. Praziquantel efficacy levels increased with drug dose. Our study showed the majority of studies reported high ERR (>80%) even among studies that reported low CR (<50%). Even in the studies reporting low CR, ERR were high indicating that treatment did reduce infection intensity even though it did not completely remove the infection in some people. Since schistosome morbidity is related to infection intensity, our results support the use of ERR as an indicator that helps avoid underestimation of praziquantel treatment efficacy and health impact.

## Introduction

Praziquantel (PZQ) has been the drug of choice for treating schistosome infection since its discovery by German pharmaceutical companies Bayer AG, Leverkusen and E. Merck, Darmstadt in 1972 [1]. To date, there is no convincing evidence of schistosomes developing resistance to PZQ in endemic areas, even in an Egyptian village where PZQ treatment has been administered for over 10 years [2], and in Zimbabwe provinces where yearly treatment have been provided for six years [3]. Previous meta-analysis studies have also concluded that PZQ is still effective against schistosome infections [4–7]. The relatively long generation time of schistosomes is thought to reduce the likelihood of these parasites developing resistance against PZQ [8–10].

Given the increase in the number of national schistosome control programs, there is still a possibility of schistosomes developing resistance against PZQ [11]. This is not a theoretical fear given the development of resistance to Oxamniquine, the drug previously used heavily to control intestinal schistosomiasis in Brazil [12]. The survival of schistosomes following PZQ treatment could result in worms developing resistance against the treatment. National schistosome control programmes rely heavily on Mass Drug Administration (MDA) [13, 14] of PZQ in populations at risk of infection (regardless of individual infection status). These MDAs may be putting selection pressure on schistosomes, leading to fears that MDAs may eventually cause the emergence of resistance [15]. In addition, PZQ has recently proved to be effective for infant and preschool children, meaning even larger populations can now be treated with PZQ [16]. These MDA programmes as well as the increasing number of studies on the safety and efficacy of PZQ in preschool children published in recent years [16], present an opportunity to conduct an up-to-date systematic review and meta-analysis to determine the factors affecting the efficacy of PZQ treatment.

PZQ treatment efficacy is normally reported as Cure Rate (CR), which compares the number of egg positive individuals pre-treatment who become negative for schistosomiasis post-PZQ treatment, as well as by the egg reduction rate (ERR), which is determined by the reduction in mean number of eggs excreted in urine (*S*. *haematobium*) or stool (*S*. *mansoni*) from pre-PZQ treatment to post-treatment. There is variability in the efficacy of praziquantel reported based on the measure used as well as from different studies [17–19]. This heterogeneity can arise from various host, parasite, or treatment regimen factors. However, to date, there has not been a comprehensive evaluation of PZQ efficacy over the past four decades and the factors that have affected the efficacy. Therefore, we conducted a meta-analysis of published PZQ efficacy studies to identify the host, parasite and treatment regimen factors that influence PZQ efficacy. The WHO recommends using ERR to measure PZQ treatment efficacy [20], through CR is still commonly used. Therefore, in this study we investigated the relationship between CR and ERR.

## Materials and methods

### Systematic review

**Fig 1:**
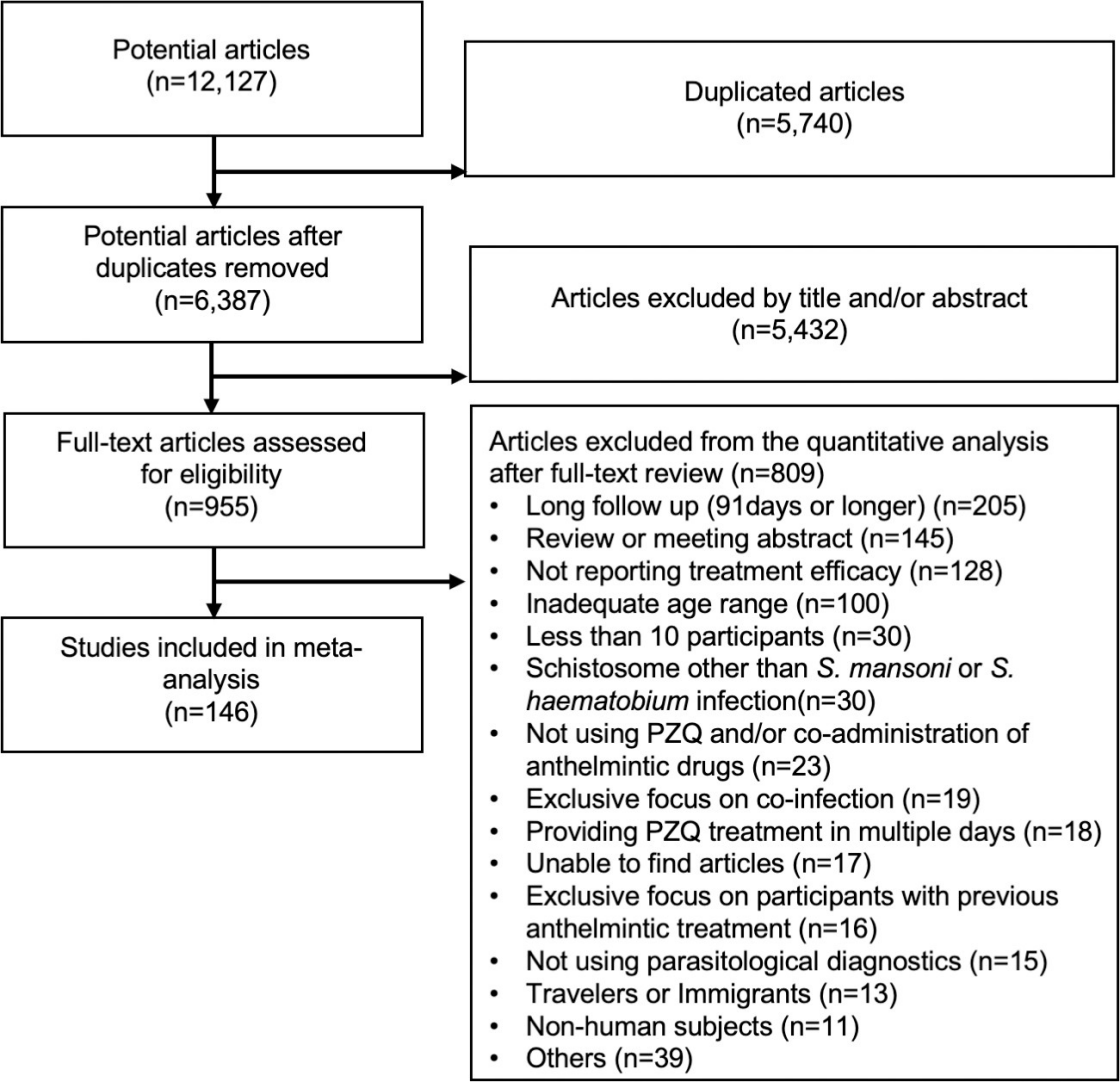
A systematic review flow diagram. Diagram of the number of articles identified and examined at each stage of the review. A total of 146 articles published from 1979 to 2020 met all inclusion criteria and were included for the meta-analysis.

In this systematic review and meta-analysis, an electronic literature search was conducted using Web of Science (www.webofknowledge.com), EMBASE (www.elsevier.com), Ovid MEDICINE (www.ovid.com), and PubMed (pubmed.ncbi.nlm.nih.gov). The search terms were; “schistosom*” AND “praziquantel” AND (“treatment” OR “efficacy” OR “cure” OR “egg reduction rate” OR “chemotherapy”). This search was completed on 15^th^ October 2020. Duplicate articles were removed, after which the titles and abstracts were reviewed as a first screening (Fig 1). Then the full texts of the remaining 955 potentially relevant articles were reviewed. Full texts of the relevant articles were sourced through the Web of Science, the Ovid, the Google Scholar (scholar.google.com), library or the Inter Library Loan of the University of Edinburgh or the University of Tsukuba. In case we could not find an article through these sources, we also contacted study authors by e-mail to request articles. Non-English articles were included, and several Chinese, French, German, Italian, Portuguese, Russian, and Spanish articles were translated into English by native speakers of each language to enable review of these publications in this study. These translations were double checked using google translate (https://translate.google.co.uk/). In addition, Japanese articles were included and reviewed without translation. All articles were reviewed by titles and abstract and/or full text by a reviewer (MF). In addition, a randomly selected subsample (240 articles) was reviewed by an independent reviewer (Catriona Waugh, the University of Edinburgh) to confirm review results from MF. The protocol of the review was as described in the materials and methods section.

A study was considered eligible if it met all of the following inclusion criteria: 1) involved human participants, 2) was based on *Schistosoma mansoni* or *S*. *haematobium* infections, 3) all participants were treated with PZQ, 4) reported the CR and/or ERR and/or schistosomiasis prevalence both before and after PZQ treatment and/or schistosomiasis infection intensity both before and after PZQ treatment, 5) the PZQ treatment was completed within a single day, which includes both single and multiple PZQ treatments, 6) the follow up study was conducted within 90 days after treatment, 7) provided participants’ age that could be categorised as either child (0–19 years old) or adult (≥20 years old), and 8) reported the number of participants.

Studies were excluded based on the following exclusion criteria: 1) used non-human subjects, 2) were performed *in vitro*, 3) involved fewer than 10 participants (e.g., a clinical case report), 4) targeted acute schistosomiasis cases, 5) were studies based on schistosome species other than *S*. *mansoni* or *S*. *haematobium*, 6) were studies based on mixed schistosome species infection, 7) were review articles or meeting abstracts, 8) had participants specially selected based on their being co-infected with other diseases such as HIV, malaria, or soil-transmitted helminths, 9) used a different anthelminthic drug (e.g., oxamniquine) together with PZQ, 10) reported CR and/or ERR not based on parasitological results (e.g., antibody levels), 11) had specially selected participants who had received any antihelminthic drug treatment prior to the PZQ treatment, 12) had participants that were not originally from endemic areas (e.g., travellers, foreign military), 13) had participants that were originally from endemic areas but had moved to non-endemic areas prior to the study (e.g., immigrants).

Articles often reported results from multiple separate groups of participants; for example, individuals from different villages. In these cases, results from each group were recorded as a single observation. A list of potential predictors (given in Table 1) was drawn up and information on these variables was extracted from each article. The potential predictors were selected based on their biological importance as suggested by previous studies [21]. There were two articles (three observations) that did not report PZQ treatment dose. In these cases, as all of them reported there was a single treatment, the treatment dose was inferred to be 40 mg/kg body weight based on the assumption that the study followed WHO guidelines [22]. There were 49 articles (94 observations) that did not report treatment year. In these cases the average interval between PZQ treatment and publication among articles which reported treatment year (i.e., two years) was used to estimate treatment year, and used for the analysis.

**Table 1:** List of potential predictors and their units/ description.

Variable name	Units/ code/ range	Range
Treatment dose	Praziquantel dose in mg/kg body weight	10–60 mg/kg body weight
Schistosome parasite species	*S*. *mansoni*, *S*. *haematobium*	Two categories
Age	Child (0–19 years old), Adult (≥20 years old)	Two categories
Time between treatment and follow up	Time between the praziquantel treatment and follow up in days	14–90 days
Treatment year	Year in which the subjects were treated with praziquantel	1977–2018
Country	Name of the country where study was conducted	26 countries
Number of days parasitological sample collected	Number of days	1–5 days
Number of parasitological samples* used for a diagnosis	Number of samples	1–12 samples

* Number of Kato Katz or urine filtration test conducted for the diagnosis.

### Statistical analysis

Random effect meta-regression models with adjusted sums of squares were used for CR and ERR separately. Multiple observations (1–20), for example CR by villages, schools were recorded from single articles and therefore each article was included as a random effect in the models. The models were built using a backwards stepwise procedure with eight potential predictors to identify statistically significant predictors (Table 1). Although use of the levels of precision of each study, such as a standard error of CR and/or ERR, is the most common weighting method for meta-regression [23], many studies in our dataset failed to report either confidence intervals, standard errors, or standard deviations of CR and/or ERR. Instead of attempting to estimate these missing values, the size of the studies (the number of participants for each observation) was used for weighting.

The influence of pre-treatment infection intensity on CR and ERR was examined by random effects meta-regression models with sequential sums of squares. Studies that treated participants with 40mg/kg body weight PZQ and reported pre-treatment infection intensity in arithmetic or geometric means were selected for this analysis (89 articles, 163 observations published between 1979 and 2020). The schistosomes species (*S*. *mansoni* or *S*. *haematobium*) and the type of mean that was used to report pre-treatment infection intensity (arithmetic mean or geometric mean) were included in the model together with pre-treatment infection intensity to control the influence of these variables.

Furthermore, the association between ERR and CR was also explored as a sub-group analysis using studies that treated participants with 40mg/kg body weight PZQ and reported both ERR and CR (87 articles, 163 observations published between 1981 and 2020) with random effect meta-regression models. For all regression models above, the outcome variable ERR was transformed as log_10_ (101- ERR) to reduce the skewness of residuals.

### Quality assurance of studies

Quality assurance of studies was conducted in two ways: 1) by using a graded scale [24], and 2) by excluding studies that failed to report the number of parasitological samples used for diagnosis. The graded scale consisted of seven criteria; i) population sampling method was described, ii) PZQ treatment was reported, iii) laboratory diagnostic methods were described, iv) pre-treatment infection intensity was reported, v) samples were collected on multiple days from each participant, vi) parasitological diagnostics were conducted twice or more for each sample, vii) types of mean to calculate CR and/or ERR was reported. Each study was given a score of one when it met a criterion and zero if it did not. The scores of seven criteria were added up and the quality of a study was categorized as follows: low 0 to 2, medium 3 to 5, high 6 to 7. Following these criteria, we identified 10 low quality articles, 17 observations published between 1981 and 2006 (S2 Appendix). Besides this, studies that did not report the number of parasitological samples used for diagnosis were also considered as low-quality studies (12 articles, 20 observations published between 1981 and 2016).

The influence of these low-quality studies on statistical analysis results was investigated by including/excluding them in separate models for 1) and 2). The analysis results showed that low-quality studies had negligible effect and therefore they were retained in our review results and statistical analyses.

### Statistical software

Articles identified by the systematic review were recorded using Thomson Reuters EndNote and the extracted data were entered in a spread sheet using Microsoft Excel 2016. *B*. *Tummers*, *DataThief III*. *2006 (**https*:*//datathief*.*org/**)* was used to extract data from published graphs. IBM SPSS Statistics Version 25.0 was used for the random effect meta-regression analysis. Microsoft Excel 2016 was used for graphical presentation.

## Results

### Systematic review results

**Fig 2:**
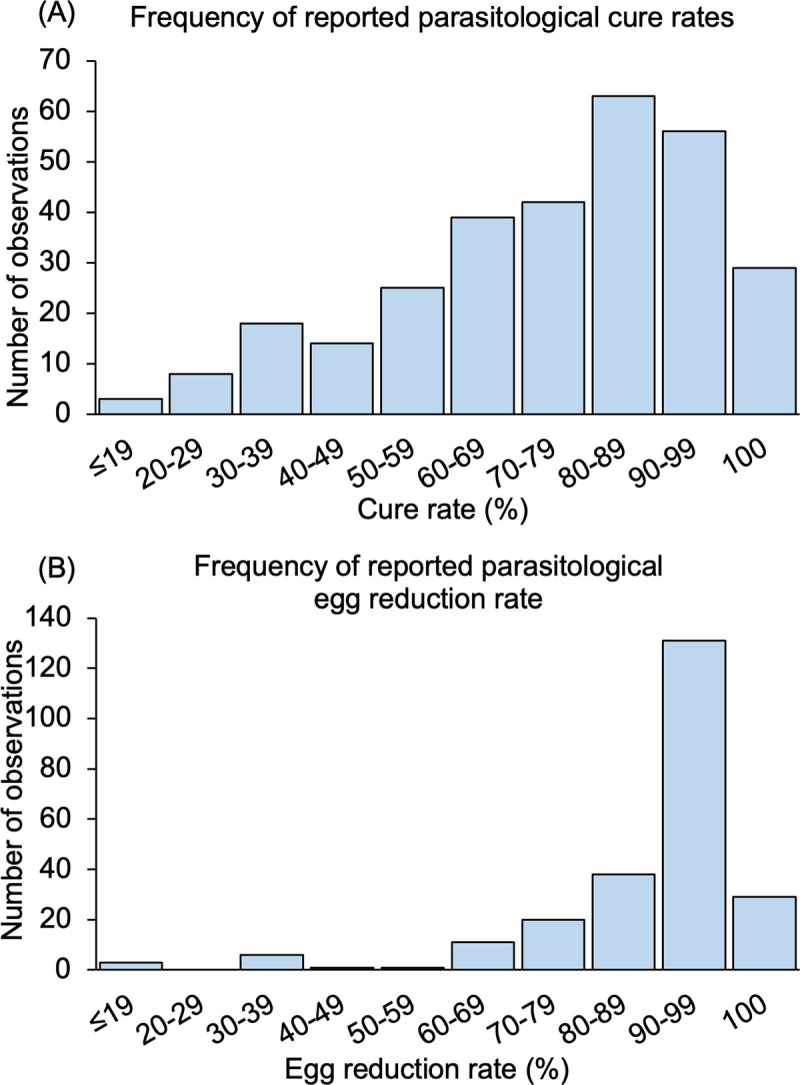
The distribution of reported parasitological cure rates and egg reduction rates following praziquantel treatment for (A) cure rate, 297 observations from 132 articles and (B) egg reduction rate, 240 observations from 108 articles.

Through an electronic literature search, 12,127 articles were identified, of which 955 articles were screened by full text. A total of 146 eligible articles published from 1979 to 2020 met all inclusion criteria and were included in the meta-analysis (Fig 1, articles are listed in S1 Appendix). A total of 325 observations were extracted from the 146 articles. The number of observations reported by a single article ranged from 1 to 20 observations. CR and ERR reported by these articles ranged from 14.3% to 100% and -20.6% to 100% respectively (Fig 2). There was one study that reported negative ERR following PZQ treatment (-20%); remaining studies reported positive ERR. The vast majority of studies (83%) reported high (>80%) ERR whereas CR showed high variability (Fig 2).

The reported PZQ doses ranged from 10 to 60 mg/kg body weight (Table 1). The majority of the studies (79%, 258 observations from 134 articles) reported the use of a treatment dose of 40 mg/kg body weight, the current WHO recommended treatment dose [25]. Although overall reported PZQ treatment doses ranged between 10–60 mg/kg body weight, studies with *S*. *mansoni*, and studies of adults, reported narrower treatment dose ranges (20–60 mg/kg body weight, 20–40 mg/kg body weight respectively). More studies were conducted with children (86%, 280 observations from 130 articles) than with adults. The reported time between treatment and follow up ranged from 14 to 90 days after PZQ treatment. Twenty-eight and 90 days were the most common time intervals between treatment and follow up (42 observations and 47 observations respectively).

### Factors affecting CR and ERR

Of the eight potential predictors (Table 1), four were found to have a significant effect (p<0.05) on the response with respect to CR: treatment dose [F(1, 249) = 10.620, p = 0.001], number of parasitological samples used for diagnosis [F(1, 104) = 6.854, p = 0.010], age category [F(1, 263) = 4.581, p = 0.033], and schistosome parasite species [F(1, 149) = 4.102, p = 0.045] (Table 2 A). Treatment dose was the only significant predictor (p<0.05) on ERR [F(1, 176) = 5.168, p = 0.024] (Table 2B).

**Table 2:** Results from random effect meta-regression models. Table shows F-values, degrees of freedoms (in parenthesis), and p-values from random effect meta-regression using adjusted sums of squares. A) four predictors were found to have a significant effect (p<0.05) on the response cure rate using backward stepwise selection; similarly, B) one predictor was found to have a significant effect (p<0.05) on the response egg reduction rate using backward stepwise selection.

A)			
Predictors	Range	F-value (df)	p-value
Treatment dose	10–60 mg/kg body weight	10.620 (1, 249)	0.001
Number of parasitological samples used for a diagnosis	1–12 samples	6.854 (1, 104)	0.010
Age	Child (0–19 years old) vs. Adult (≥ 20 years old)	4.581 (1, 263)	0.033
Schistosome parasite species	*S*. *mansoni* vs. *S*. *haematobium*	4.102 (1, 149)	0.045
B)			
Predictors	Range	F-value (df)	p-value
Treatment dose	10–60 mg/kg body weight	5.168 (1, 176)	0.024

**Fig 3:**
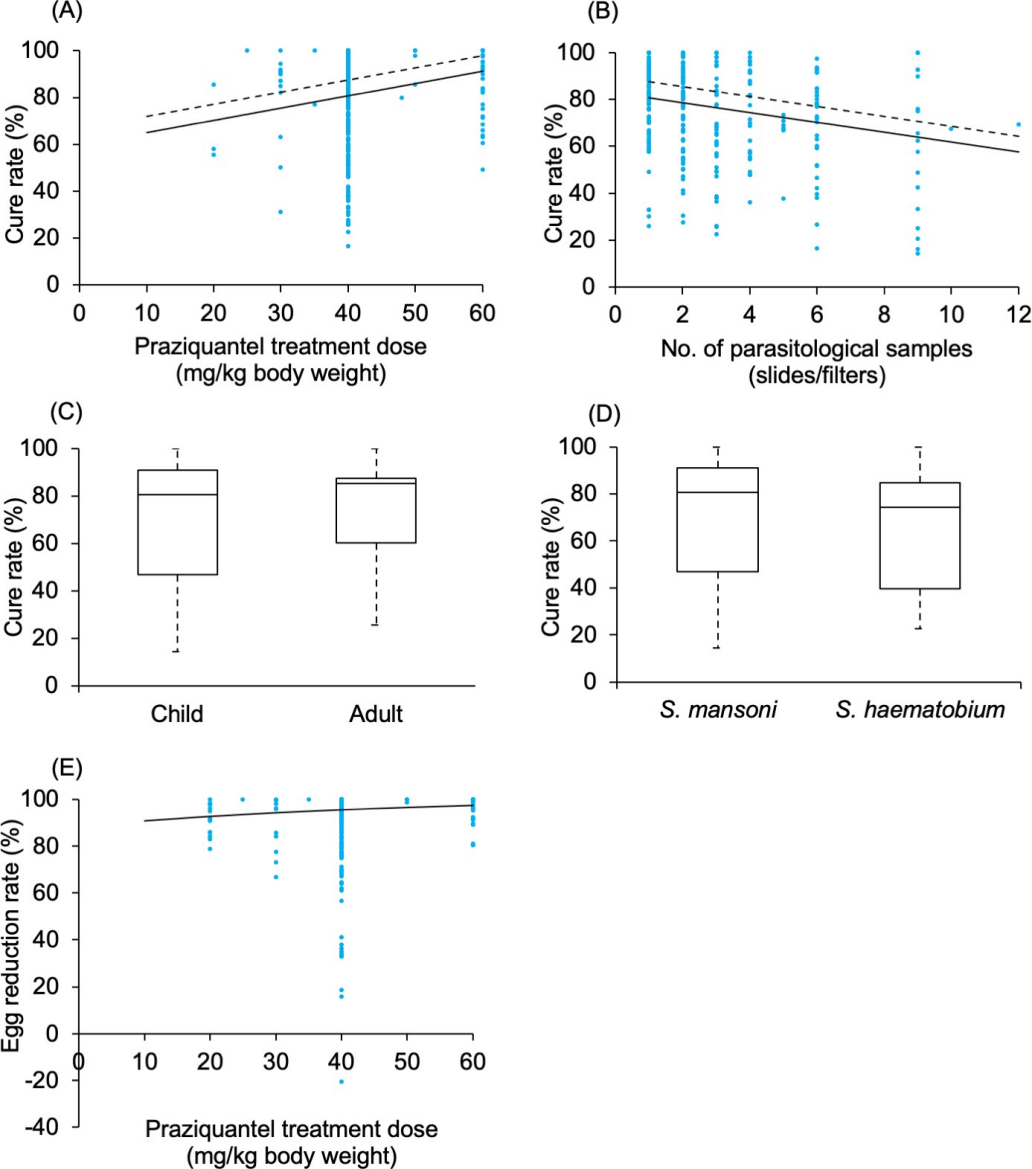
Fitted graphs for predictors identified from a random effects meta-regression model. Identified predictors’ effects on cure rate (graph A, B, C and D) and egg reduction rate (graph E): (A) praziquantel treatment dose over the range 10–60 mg/kg body weight on cure rate (B) the number of parasitological samples used for a diagnosis over the range 1–12 samples on cure rate (C) age category child (0 to 19 years old) and adults (20 years old or older) (D) parasite species *S*. *mansoni* and *S*. *haematobium* (E) praziquantel treatment dose over the range 10–60 mg/kg body weight on egg reduction rate. **Scatter plot graphs A, B, and E**: Data points indicate reported cure rate/egg reduction rate for each observation. Negative fraction indicates that praziquantel treatment was associated with increase of schistosome infection intensity. Lines are fitted graphs generated from random effects meta-regression. **Scatter plot graph A and B**: Dashed lines denote the highest level of cure rate over range that could be achieved among adults with *S*. *mansoni* infection treated with 60 mg/kg body weight praziquantel (for graph B) and one parasitological sample used for a diagnostic (for graph A). Heavy lines denote the cure rate over the reported dose/number of samples using mode of other predictors, i.e., children infected with *S*. *mansoni* and treated with 40 mg/kg body weight praziquantel treatment dose, diagnosed by one parasitological sample. **Box plot C and D**: Boxes denote range of possible maximum and minimum cure rate (%) estimated by models with heavy lines denoting model estimated cure rate using mode of predictors. Dashed lines denote the range of raw reported cure rate by each observation.

The model results indicated a positive relationship between PZQ treatment dose for both CR and ERR (Fig 3A and 3E). The model results showed a negative relationship between the number of parasitological samples (slides/ filters) and CR (Fig 3B). The model results suggested a higher CR for *S*. *mansoni* infection than *S*. *haematobium* infection, and a higher CR in adults than children (Fig 3C and 3D). The model results revealed that the current WHO recommended dose (40 mg/kg body weight) could achieve 95% ERR, and 57% to 88% CR with the outcome depending on schistosome parasite species, age of participants, and number of parasitological samples used for diagnosis.

**Fig 4:**
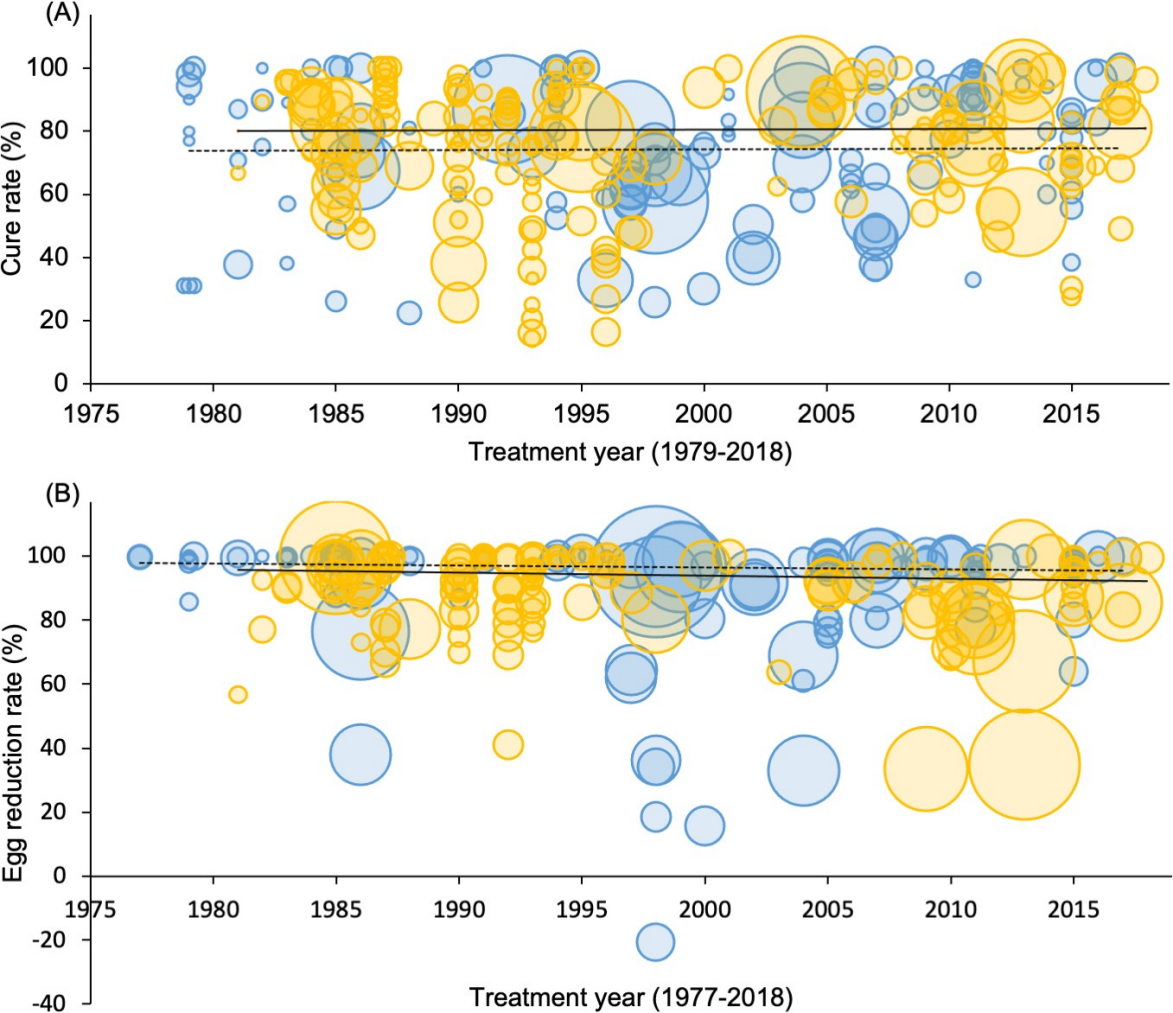
Scatter graphs of the reported cure rate (graph A) and egg reduction rate (graph B) by treatment year. Each plot represents the reported cure rate/egg reduction rage of *S*. *haematobium* in blue and *S*. *mansoni* in orange. The size of each plot represents the number of participants of each observation. Lines are fitted graphs generated from random-effects meta-regression models. Heavy lines denote the cure rate or egg reduction rate of *S*. *mansoni* infection. Dashed lines denote the cure rate and egg reduction rate of *S*. *haematobium* infection. For graph A: fitted lines denote the cure rate over the reported treatment years that could be achieved among children treated with 40 mg/kg bodyweight praziquantel and one parasitological sample used for a diagnostic. Similarly, for graph B, fitted lines denote the egg reduction rate with 40 mg/kg body weight praziquantel treatment.

The model results also showed that the treatment year does not have a statistically significant effect on CR [F(1, 117) = 0.018, p = 0.894] when it was included in the model together with statistically significant four predictors (treatment dose, number of parasitological samples used for diagnostics, age category, and schistosome parasite species). Similarly, the treatment year did not show a statistically significant influence on ERR [F(1, 109) = 1.917, p = 0.169] when it was included in the model together with treatment dose (Fig 4A and 4B).

**Fig 5:**
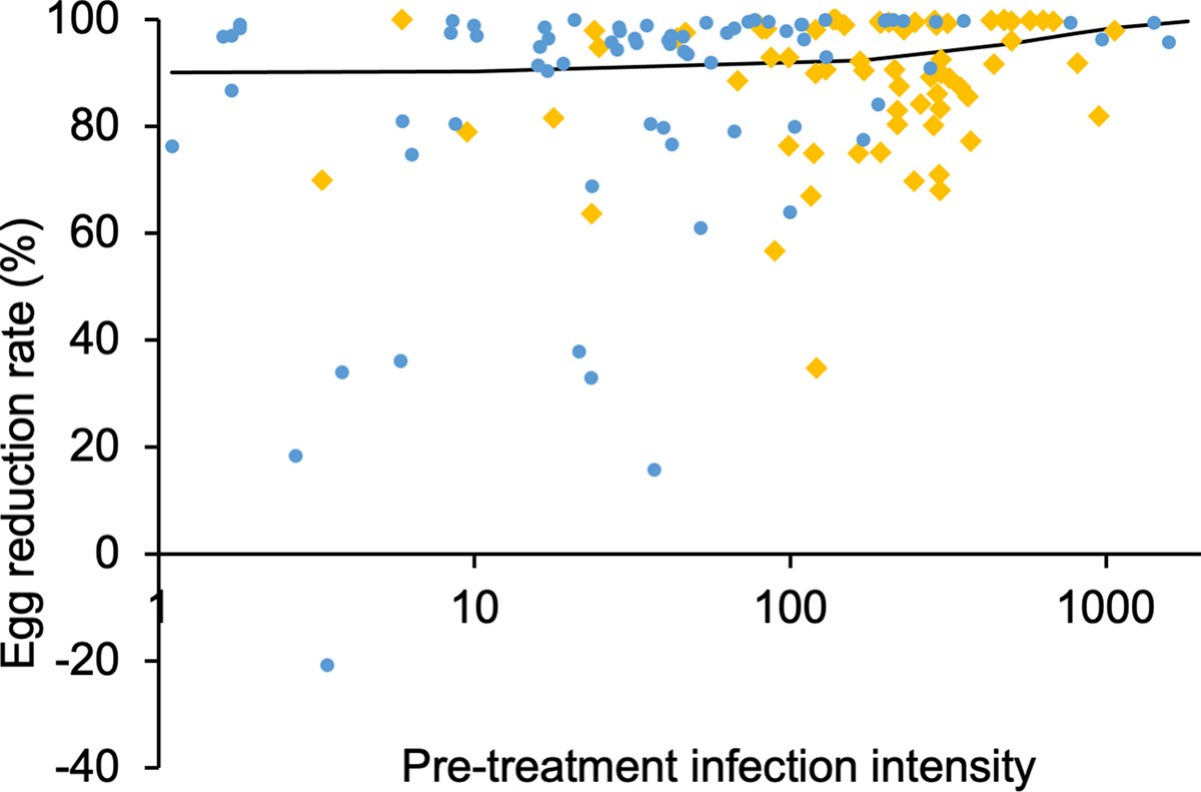
Scatter graph of the reported egg reduction rate in percentage vs pre-treatment infection intensity (eggs/10ml urine for *S*. *haematobium* in blue circle and eggs/g of feces for *S*. *mansoni* in orange diamond). The fitted line is from a random effects meta-regression model which confirmed statistically significant association between pre-treatment infection intensity and egg reduction rate [F = 11.257 (1, 118), p = 0.001]. For this model both schistosome species and type of pre-treatment infection intensity mean (arithmetic and geometric mean) were considered. A total of 134 observations from 70 articles were used for this analysis.

The relationship with pre-treatment infection intensity and CR or ERR was analyzed separately using two independent random effect meta-regression models. The model results indicated a positive relationship between pre-treatment infection intensity and ERR [F = 11.257 (1, 118), p = 0.001] (Fig 5). There was no statistically significant relationship between pre-treatment infection intensity and CR.

**Fig 6:**
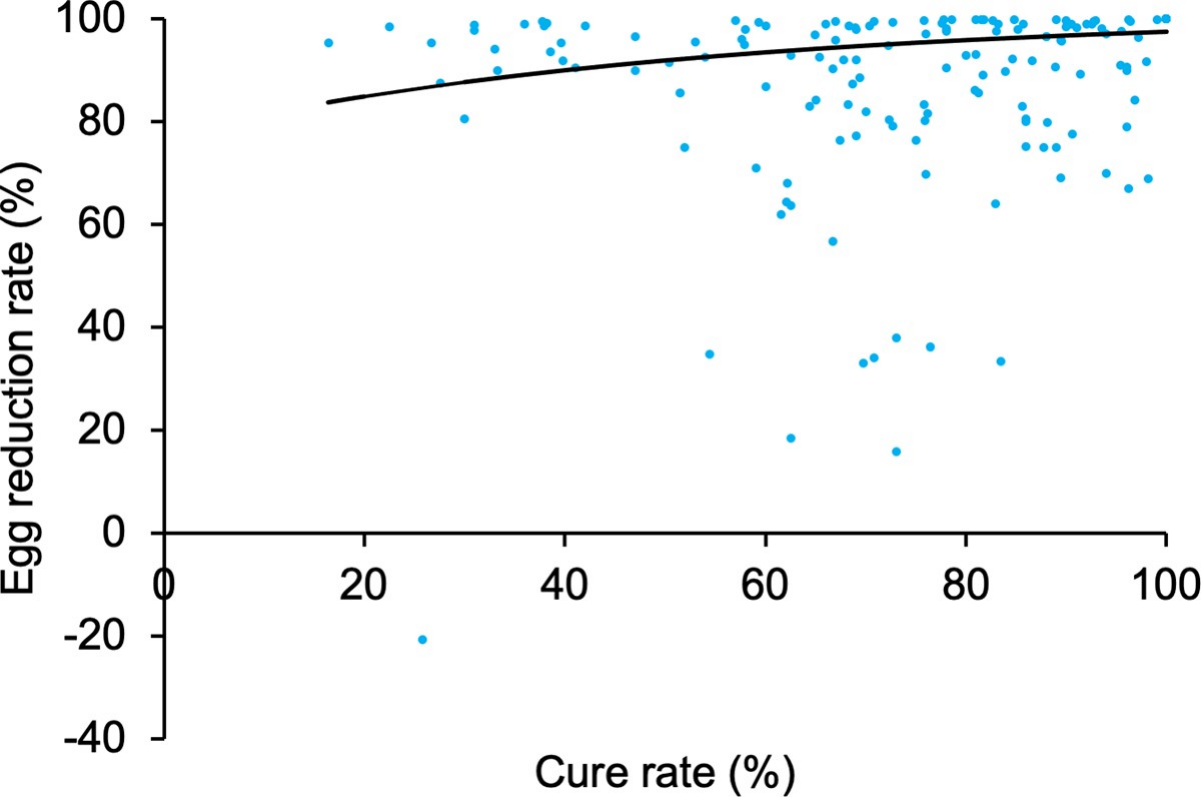
Scatter graph of the reported egg reduction rate in percentage (%) vs cure rate in observations that used 40 mg/kg body weight praziquantel treatment dose (163 observations from 87 articles). The fitted line is from a random effects meta-regression model which confirmed statistically significant association between cure rate and egg reduction rate [F = 20.097 (1, 147), p<0.001].

The random effect meta-regression analyses results indicated a positive relationship between CR and ERR [F = 20.097 (1, 147), p<0.001]. The model estimated high ERR (84% to 97%) over the reported CR (16% to 100%) (Fig 6). Similarly, even in those studies that reported low (50% or lower) CR the reported ERR was high (>80%) except one study that reported an ERR of -20%.

## Discussion

We conducted a meta-analysis of the PZQ efficacy level using published articles to identify predictors that have any influence on CR and/or ERR. The results confirm that PZQ has sustained its efficacy in treating schistosome infection over four decades of usage. This study indicated that low CR and ERR could be improved by higher PZQ treatment doses. Furthermore, the analyses revealed that over the reported treatment years (1977 to 2018) there was considerable variability in CR. ERR was more consistent. Nonetheless, there was a statistically significant positive relationship between CR and ERR. The majority of individual studies also reported a high ERR (>80%) even among studies that reported low CR (<50%). A single egg in the parasitological sample counts as failure of cure even if there has been a considerable reduction in infection intensity leading to low CR but high ERR. This highlights the complication of using CR, which may lead to the underestimation of treatment efficacy.

There was no significant effect of treatment year on CR or ERR, which suggests PZQ efficacy remained stable over the 40 years from 1977 to 2018. Results also indicated that the current WHO recommended treatment dose (40 mg/kg body weight) can achieve 95% ERR regardless schistosomes species and 57% to 88% CR depending on schistosomes species, host age, and number of parasitological samples. This finding of the efficacy of the current standard treatment dose (40 mg/kg body weight) is consistent with the findings of recently published systematic reviews. Systematic reviews conducted in 2015 and 2017 have reported high efficacy of PZQ treatment with standard dose in pre-school and school aged children for *S*. *mansoni* and/or *S*. *haematobium* infection [6, 7]. Based on these findings, there was no evidence of schistosomes developing resistance and/or tolerance against PZQ treatment from this study. Nevertheless, there is a need for sustained monitoring and evaluation of PZQ efficacy and parasite drug sensitivity as the number of programs undertaking preventive chemotherapy for schistosomiasis are increasing. For example, a number of countries (Egypt, Kenya, Senegal, Togo, Burkina Faso, Nigeria, Cameroon, Burundi, and Zimbabwe) have had sustained MDAs for over 5 years [3, 14, 26, 27].

Our results showed that the CR and ERR of PZQ increased with dose over the range of 10–60 mg/kg body weight. Supporting this result, Taylor *et al*. previously reported a similar increase of CR with an increase in the PZQ doses over 10–40 mg/kg body weight both for *S*. *mansoni* and *S*. *haematobium* infection [28]. Our results might suggest that a higher PZQ dose than the current recommendation (40 mg/kg body weight) could potentially improve the treatment efficacy. However, the absence of studies for adults treated with an elevated PZQ dose (>40 mg/kg body weight) made it impossible to estimate the effect of a higher dose in adults. In addition, the risk of having adverse events after PZQ treatment has been reported to be higher after elevated doses are administered (>40 mg/kg body weight) [29] compared to the lower frequency of adverse events in people receiving dosages of 40 mg/kg body weight or lower [30]. In the case of higher PZQ doses, treatment efficacy has to be weighed against the risk of adverse reactions, in areas of persistent infection to achieve better CR and ERR [31, 32].

In this analysis, we selected studies where the full dose of PZQ was administered in a single day as opposed to over multiple days. This was due to the high heterogeneity in the number of treatments and the days between the treatments among studies administering multiple treatments over different days. Among the excluded studies Tchuenté *et al*. [33] reported high CR (>95% for *S*. *mansoni* infection, >80% for *S*. *haematobium* infection) after PZQ treatment using a total dose of 80 mg/kg body weight, which was administrated in two treatments over a 3 weeks interval. Similarly, N’Goran *et al*. [34] reported high *S*. *haematobium* infection CR (>90%) after the two oral doses of PZQ (each of 40mg/kg body weight) 4 weeks apart. Furthermore, the same study reported that significantly lower adverse events occurred after the 2^nd^ PZQ treatment in comparison to the 1^st^ treatment [34]. These reports indicate that higher treatment efficacy can be achieved with increased doses administered over several days. While this also reduces the likelihood of adverse events, it risks reduced compliance during follow up treatments meaning people may not complete the full treatment course.

Only a few studies reported both CR and post-treatment infection intensity; and using these data we were able to demonstrate that the majority of studies reporting low CR also reported very low posttreatment infection intensity, giving a high ERR. This is not surprising. In general, the majority of studies reported a high ERR (>80%) even among studies that reported low CR (<50%). Our model results also estimated a high ERR (84% to 97%) over the reported range of CR (16% to 100%). Schistosome morbidity is related to parasite egg intensity [35], and the efficacy of PZQ in reducing parasite burden supports the use of ERR as recently recommended by WHO for evaluating PZQ efficacy [20].

Our results suggest a significantly higher CR in adults (≥20 years old) than in children (0–19 years old). In a previous meta-analysis, Stothard *et al*. [21] have reported that there was a negligible difference between pre-school children and school aged children in their CR levels, suggesting that the effect of host age on PZQ efficacy levels takes time to become detectable. The CR difference between adults and children could be due to a difference in the schistosome-specific protective immunity levels difference between them. The protective immunity level is known to be higher among adults in endemic areas and that could improve the PZQ treatment efficacy levels [36, 37]. Supporting this, enhanced cure rates have been reported in experimental mice with high levels of schistosome parasite specific antibodies [38, 39]. Besides host protective immunity against schistosome infection, it is also likely that there are pharmacokinetic differences between adults and children. Supporting this, Kovac *et al*. have reported differences in PZQ pharmacokinetics between school-aged and preschool-aged children infected with *S*. *mansoni* [40]. However, there have not yet been comparative studies between adults and children. An even less studied aspect of PZQ efficacy is host pharmacogenetics. Such studies, together with parasite population genetic studies will elucidate other potential influence on PZQ efficacy.

Some predictor variables were significant for CR, but not for ERR, including the age of participants, the schistosome species and the number of parasitological samples examined. The standard parasitological diagnostic methods for both *S*. *mansoni* and *S*. *haematobium* infection (Kat Katz, and urine filtration respectively) have low sensitivity particularly for participants with low infection intensity [41–44]. This could influence the estimated CR difference found between *S*. *mansoni* and *S*. *haematobium* infection, as parasite eggs may be more easily detected in urine samples than faecal samples after praziquantel treatment. Our modelling results showed a higher CR of *S*. *mansoni* infection than *S*. *haematobium* infection. Furthermore, diagnostic sensitivity is known to increase with an increase in the number of parasitological samples used for the diagnosis [45] which was significant for CR but not ERR in our analysis. This could be because the detection of a single egg can make the difference between cured and non-cured: the impact of diagnostic sensitivity is larger on CR than on ERR. Together, our results may suggest that the sensitivity of parasitological diagnostic tests influences ERR less than CR. Lamberton *et al*. have suggested using at least four Kato-Katz tests for highly endemic areas and six Kato-Katz tests for low endemic areas to achieve reasonable sensitivity [45]. Nevertheless, the majority of studies included in our study used only one or two Kato-Katz or urine filtrations for the diagnosis (171 observations, 57%). Considering current situations, using ERR has an advantage as it would be less affected by the sensitivity of parasitological diagnostic tests.

Our analyses did not demonstrate a significant influence of the time between praziquantel treatment and follow up on CR and ERR within the reported follow up days (14–90 days). However, care is needed in interpretation of this result, because both shorter or longer follow up times could cause inaccurate CR and ERR for different reasons, which were not identifiable in our study. Short follow-up intervals after PZQ treatment are thought to increase the risk of the contamination by dead eggs which reduces recorded CR and ERR [18, 32, 46–48], as it is difficult to differentiate live eggs from dead ones in parasitological diagnostic procedures [18, 46]. Thus, although Guidi *et al*. reported low cure rates after praziquantel treatment (46%), they also reported that almost all the eggs (>95%) detected in the post-treatment samples were dead [47]. On the other hand, a long follow-up time could increase the risk of re-infection, and also the influence of immature worms which may have survived the treatment. Both could decrease CR and ERR. Praziquantel treatment is more effective on adult schistosome worms than on immature ones [49, 50], therefore there is a possibility of participants being categorized as non-cured even when praziquantel treatment successfully clear adult worms; if participants were infected with immature worms. As such, an influence of egg contamination is smaller in ERR than CR, thus predicating the use of ERR especially in highly endemic areas. There are studies which have concluded that praziquantel treatment was still effective because of a high ERR after the treatment, despite low CR [48, 51, 52]. Therefore, measuring ERR could be advantageous for better understanding the impact of praziquantel treatment on schistosomiasis burden, especially in high transmission areas.

Our analyses did not detect any significant influence of country on CR and ERR. Although low cure rates (<50%) have been reported from different African countries over the years, high cure rates (>90%) have also consistently been reported from different areas of the same countries. These results again suggest that PZQ treatment still maintained its effectiveness for schistosome infection. Nevertheless, there is still a risk of missing the parasites acquiring resistance against PZQ treatment within each country. This is because the majority of these studies were conducted in different study areas (e.g., different villages) within the same country. Schistosomiasis transmission is known to be focal in endemic areas as transmission is regulated by the distribution of intermediate fresh water snail hosts. Therefore, disease transmission could vary even within a given endemic area depending on the natural water sources that people use for their daily lives [53, 54]. A heavy schistosomiasis burden, which can be indicated by high infection prevalence, has been reported to reduce PZQ CR [21]. Nevertheless, in our analyses, it was impossible to distinguish between the influence of treatment year and study area on CR and ERR within each country. This is because even studies were conducted in the same countries, these normally include multiple different study areas. Long term cohort studies in areas undergoing mass drug administration programmes will therefore be important for detecting any reduction of PZQ efficacy levels.

There are a number of factors that could not be included in the current study, regardless of their potential to influence on PZQ efficacy, due to the small number of studies that addressed them. For example, the source and manufacturer of PZQ is a potential confounder. Variation in generic PZQ quality has been reported with fake PZQ having been used in some countries [55, 56]. Thus, PZQ quality may have impacted treatment efficacy. Providing a snack or drink prior to PZQ treatment is reported to improve the drug’s efficacy [57]. Some studies reported providing a snack and/or juice before the PZQ treatment [29, 37, 57–64], or to have treated participants after their breakfast, lunch or dinner [65–67]. However, this could not be taken into account in this analysis as the majority of the studies did not report on supplemental feeding. It has also recently been reported that host pharmacokinetic factors influence treatment efficacy [68]. However, this could not be included in this analysis as host pharmacokinetic factors have not been reported by most studies.

Schistosomiasis infection intensity and related morbidity have been reported to vary among individuals, even in populations within the same geographical areas (e.g., villages, neighbours sharing the same water sources). This could be due to the variation of water-contact frequencies [69], age [70], or the levels of acquired immunity [70, 71]. However, as there was no available data for individual cases in the majority of studies, the mean values of the participants were used both for response variables (CR and ERR) and predictors. A group of participants often consisted of individuals of different age, co-infection status with other pathogens, and with different levels of schistosomiasis burden. Therefore, extra care must be taken when translating the results from meta-analysis into real epidemiological situations. When publishing the raw study data becomes more common in the field of epidemiology, more detailed meta-analyses could be undertaken by synthesizing individual-level raw data from multiple studies. This would enable an increase in the number of potential predictors, and could also enhance the general applicability of the findings from the analyses.

## Conclusion

Our analyses confirmed the efficacy of PZQ treatment for schistosome infection regardless of decades of intensive usage of the drug worldwide. CR were higher among adults compared to children and in *S*. *mansoni*- than *S*. *haematobium*-infected people. PZQ efficacy increased with increasing drug dose over the range of 10–60 mg/kg body weight with a single time administration. Conversely, CR decreased with increasing number of diagnostic samples. Although there was a positive association between CR and ERR, the estimated ERR was high (>80%) even among studies that reported low CR (<50%). As schistosome parasite eggs are the main cause of schistosomiasis pathology, the reduction of infection intensity after treatment is a better measure of the impact of treatment on human health and justifies the WHO recommendation of using ERR for measuring PZQ efficacy. Overall, our study indicates that PZQ efficacy has not declined despite the increased use in national MDAs. There is now a need for more studies e.g., pharmacokinetic and ecological studies to identify ways in which we can further improve efficacy in areas reporting lower ERR [72].

## Supporting information

S1 Appendix List of articles included for the analysis [1–146]. (DOCX)

S2 Appendix Quality assurance of studies. When a study met and/or reported each criterion a score of one was given. A study categorized “high quality” when a total score was 6–7, “medium” with 3–5, and “low” with 0–2.
(DOCX)
