# Supplementary material for: Efficacy of praziquantel has been maintained over four decades (from 1977 to 2018): A systematic review and meta-analysis of factors influence its efficacy
Source: PLoS Negl Trop Dis. 2021 Mar 17;15(3):e0009189. doi: 10.1371/journal.pntd.0009189 (PMC7968639; doi:10.1371/journal.pntd.0009189)
Supplement: S1 Appendix — (DOCX) [file pntd.0009189.s001.docx]

**Appendix 1. List of articles included for the analysis [1-146]**

1. Abu-Elyazeed RR, Mansour NS, Habib M, Podgore JK. *Schistosoma mansoni* infection 3 months after praziquantel therapy among farmers in Qalyub, Egypt. J Trop Med. 1993;2(4):3-7.

2. Abu-Elyazeed RR, Mansour NS, Youssef FG, Boghdadi AM, el Khoby TA, Hassanein YA, et al. Seasonality as a determinant of the efficacy of praziquantel in population-based chemotherapy: lessons from the practice. J Egypt Soc Parasitol. 1998;28(1):1-7.

3. Adoubryn KD, Kouadio-Yapo CG, Ouhon J, Aka NAD, Bintto F, Assoumou A. Intestinal parasites in children in Biankouma, Ivory Coast (mountaineous western region): efficacy and safety of praziquantel and albendazole. [French]. Med Sante Trop. 2012;22(2):170-6.

4. Alharbi RA, Alwajeeh TS, Assabri AM, Almalki SSR, Alruwetei A, Azazy AA. Intestinal parasitoses and schistosome infections among students with special reference to praziquantel efficacy in patients with schistosomosis in Hajjah governorate, Yemen. Annals of Parasitology. 2019;65(3):217-23.

5. Anwar WA, Rosin MP. Reduction in chromosomal damage in schistosomiasis patients after treatment with praziquantel. Mutat Res. 1993;298(3):179-85.

6. Augusto G, Magnussen P, Kristensen TK, Appleton CC, Vennervald BJ. The influence of transmission season on parasitological cure rates and intensity of infection after praziquantel treatment of *Schistosoma haematobium*-infected schoolchildren in Mozambique. Parasitology. 2009;136(13):1771-9.

7. Bajiro M, Dana D, Ayana M, Emana D, Mekonnen Z, Zawdie B, et al. Prevalence of *Schistosoma mansoni* infection and the therapeutic efficacy of praziquantel among school children in Manna District, Jimma Zone, southwest Ethiopia. Parasit Vectors. 2016;9(1):560.

8. Banwat ME, Daboer JC, Envuladu EA, Lar LA, Ogbonna CC. Effect of mass chemotherapy with praziquantel on the prevalence of schistosomiasis in school children in Langai community of Plateau State. J Med Trop. 2011;13(2):119-23.

9. Barakat R, El Morshedy H. Efficacy of two praziquantel treatments among primary school children in an area of high *Schistosoma mansoni* endemicity, Nile Delta, Egypt. Parasitology. 2011;138(4):440-6.

10. Barda B, Coulibaly JT, Puchkov M, Huwyler J, Hattendorf J, Keiser J. Efficacy and safety of moxidectin, synriam, synriam-praziquantel versus praziquantel against *Schistosoma haematobium* and *S. mansoni* infections: A randomized, exploratory phase 2 trial. PLoS Negl Trop Dis. 2016;10(9):e0005008.

11. Ben SA, Useh MF. A comparative study on the efficacy of praziquantel and albendazole in the treatment of urinary schistosomiasis in Adim, Cross River State, Nigeria. Int Health. 2017;9(5):288-93.

12. Black CL, Steinauer ML, Mwinzi PNM, Secor WE, Karanja DMS, Colley DG. Impact of intense, longitudinal retreatment with praziquantel on cure rates of *Schistosomiasis mansoni* in a cohort of occupationally exposed adults in western Kenya. Trop Med Int Health. 2009;14(4):450-7.

13. Borrmann S, Szlezak N, Faucher JF, Matsiegui PB, Neubauer R, Binder RK, et al. Artesunate and praziquantel for the treatment of *Schistosoma haematobium* infections: A double-blind, randomized, placebo-controlled study. J Infect Dis. 2001;184(10):1363-6.

14. Botros S, Sayed H, El-Dusoki H, Sabry H, Rabie I, El-Ghannam M, et al. Efficacy of mirazid in comparison with praziquantel in Egyptian *Schistosoma mansoni*-infected school children and households. Am J Trop Med Hyg. 2005;72(2):119-23.

15. Burchard GD, Kern P, Baltes R, Dietrich M. Comparative trial of oltipraz versus praziquantel in the treatment of urinary schistosomiasis in the gabon. Tropenmed Parasitol. 1984;35(2):91-4.

16. Bustinduy AL, Sousa-Figueiredo JC, Adriko M, Betson M, Fenwick A, Kabatereine N, et al. Fecal occult blood and fecal calprotectin as point-of-care markers of intestinal morbidity in ugandan children with *Schistosoma mansoni* infection. PLoS Negl Trop Dis. 2013;7(11).

17. Bustinduy AL, Waterhouse D, de Sousa-Figueiredo JC, Roberts SA, Atuhaire A, Van Dam GJ, et al. Population pharmacokinetics and pharmacodynamics of praziquantel in Ugandan children with intestinal schistosomiasis: Higher dosages are required for maximal efficacy. MBio. 2016;7(4).

18. Butterworth AE, Sturrock RF, Ouma JH, Mbugua GG, Fulford AJC, Kariuki HC, et al. Comparison of different chemotherapy strategies against *Schistosoma mansoni* in Machakos District, Kenya: effects on human infection and morbidity. Parasitology. 1991;103(3):339-55.

19. Cabello R, Beck L, Massara CL, Murta FLG, Guimaraes R, Pieri OS, et al. *Schistosoma mansoni* infection and related knowledge among schoolchildren in an endemic area of Minas Gerais, Brazil, prior to educational actions. Acta Trop. 2016;164:208-15.

20. Campagne G, Garba A, Barkire H, Vera C, Sidiki A, Chippaux JP. Long-term echographic monitoring of children with Schistosomiasis haematobia after praziquantel. Trop Med Int Health. 2001;6(1):24-30.

21. Chisango TJ, Ndlovu B, Vengesai A, Nhidza AF, Sibanda EP, Zhou D, et al. Benefits of annual chemotherapeutic control of schistosomiasis on the development of protective immunity. BMC Infect Dis. 2019;19.

22. Chuks Ejezie G, Okeke GC. Chemotherapy in the control of urinary schistosomiasis in Nigeria. J Trop Med Hyg. 1987;90(3):149-51.

23. Clercq Dd, Hanne C, Vercruysse J. Selected chemotherapy and seasonally transmitted *Schistosoma haematobium* infections in the middle valley of the Senegal River Basin. Trans R Soc Trop Med Hyg. 2000;94(2):198-9.

24. Clercq Dd, Vercruysse J, Kongs A, Verle P, Dompnier JP, Faye PC. Efficacy of artesunate and praziquantel in *Schistosoma haematobium* infected school children. Acta Trop. 2002;82(1):61-6.

25. Coulibaly JT, Panic G, Silue KD, Kovac J, Hattendorf J, Keiser J. Efficacy and safety of praziquantel in preschool-aged and school-aged children infected with *Schistosoma mansoni*: a randomised controlled, parallel-group, dose-ranging, phase 2 trial. Lancet Glob Health. 2017;5(7):e688-e98.

26. Coulibaly JT, Panic G, Yapi RB, Kovac J, Barda B, N'Gbesso YK, et al. Efficacy and safety of ascending doses of praziquantel against *Schistosoma haematobium* infection in preschool-aged and school-aged children: a single-blind randomised controlled trial. BMC Med. 2018;16(1):81.

27. Davis A, Biles JE, Ulrich AM, Dixon H. Tolerance and efficacy of praziquantel in phase II A and II B therapeutic trials in Zambian patients. Arzneimittelforschung. 1981;31(3a):568-74.

28. Degu G, Mengistu G, Jones J. Praziquantel efficacy against *Schistosomiasis mansoni* in schoolchildren in north-west Ethiopia. Trans R Soc Trop Med Hyg. 2002;96(4):444-5.

29. Doehring E, Ehrich JHH, Reider F. Daily urinary protein loss in *Schistosoma haematobium* infection. Am J Trop Med Hyg. 1986;35(5):954-8.

30. El-Ghandour S, El-Ayat A, Saleh M. Optimizing praziquantel dose for mass treatment of children with *Schistosoma mansoni* in Egypt: A trial to cut down the cost for developing countries. Pediatr Rev Commun. 1989;4(1-2):61-6.

31. El-Hawey AM, Abou-Taleb SA, El-Gammal HA, Abdel-Rahman MM, Sabry AA. Profile of portal hypertension in hepatosplenic schistosomiasis. J Egypt Soc Parasitol. 1992;22(2):305-9.

32. El-Morshedy H, Kinosien B, Barakat R, Omer E, Khamis N, Deelder AM, et al. Circulating anodic antigen for detection of *Schistosoma mansoni* infection in Egyptian patients. Am J Trop Med Hyg. 1996;54(2):149-53.

33. Elbasheir MM, Karti IA, Elamin EM. Evaluation of a rapid diagnostic test for Schistosoma mansoni infection based on the detection of circulating cathodic antigen in urine in Central Sudan. PLoS Negl Trop Dis. 2020;14(6).

34. Erko B, Degarege A, Tadesse K, Mathiwos A, Legesse M. Efficacy and side effects of praziquantel in the treatment of *Schistosomiasis mansoni* in schoolchildren in Shesha Kekele Elementary School, Wondo Genet, Southern Ethiopia. Asian Pac J Trop Biomed. 2012;2(3):235-9.

35. Farid L, el-Masry NA, Bassily S, Trabolsi B, Wallace CK. Treatment of bilharzial obstructive uropathy with praziquantel. J Infect Dis. 1984;150(2):307-8.

36. Friis H, Byskov J. The effect of praziquantel against *Schistosoma mansoni*-infections in Botswana. Trop Geogr Med. 1989;41(1):49-51.

37. Garba A, Tohon Z, Sidiki A, Chippaux JP, Chabalier Fd. Efficacy and tolerance of praziquantel in school-aged children in a *Schistosoma haematobium* hyperendemic area (Niger, 1999). Bull Soc Pathol Exot. 2001;94(1):42-5.

38. Grogan JL, Kremsner PG, van Dam GJ, Metzger W, Mordmuller B, Deelder AM, et al. Antischistosome IgG4 and IgE responses are affected differentially by chemotherapy in children versus adults. J Infect Dis. 1996;173(5):1242-7.

39. Groning E, Bakathir H, Salem A, Albert L, Fernandez R. Effectiveness of and tolerance to praziquantel in schistosomiasis. Rev Cubana Med Trop. 1985;37(2):215-9.

40. Gryseels B, Nkulikyinka L, Coosemans MH. Field trials of praziquantel and oxamniquine for the treatment of *Schistosomiasis mansoni* in Burundi. Trans R Soc Trop Med Hyg. 1987;81(4):641-4.

41. Gryseels B, Nkulikyinka L, Engels D. Repeated community-based chemotherapy for the control of *Schistosoma mansoni*: effect of screening and selective treatment on prevalences and intensities of infection. Am J Trop Med Hyg. 1991;45(4):509-17.

42. Guidi A, Andolina C, Makame Ame S, Albonico M, Cioli D, Juma Haji H. Praziquantel efficacy and long-term appraisal of schistosomiasis control in Pemba Island. Trop Med Int Health. 2010;15(5):614-8.

43. Guisse F, Polman K, Stelma FF, Mbaye A, Talla I, Niang M, et al. Therapeutic evaluation of two different dose regimens of praziquantel in a recent *Schistosoma mansoni* focus in northern Senegal. Am J Trop Med Hyg. 1997;56(5):511-4.

44. Gyoten J, Kimura E, Muhoho ND. *Schistosoma haematobium* reinfection occurring shortly after treatment with praziquantel. Jpn J Trop Med Hyg. 1992;20(2):157-64.

45. Hailu T, Abera B, Mulu W, Alemu M, Yizengaw E, Genanew A. Efficacy of single dose albendazole and praziquantel drugs among helminth-infected school children at Rural Bahir Dar, northwest Ethiopia. Tropical Doctor. 2018;48(4):270-2.

46. Hamm DM, Agossou A, Gantin RG, Kocherscheidt L, Banla M, Dietz K, et al. Coinfections with *Schistosoma haematobium*, *Necator americanus* and *Entamoeba histolytica/Entamoeba dispar* in children: Chemokine and cytokine responses and changes after antiparasite treatment. J Infect Dis. 2009;199(11):1583-91.

47. Hassanein HI, Zoheiry MMK, Voss B, El-Attar GM, Hassan SI, Demerdash ZA, et al. Effect of early treatment with praziquantel on serum connective tissue metabolite markers in children and adolescents with intestinal *Schistosomiasis mansoni*. Arzneimittelforschung. 1997;47(1):84-7.

48. Hatz CF, Vennervald BJ, Nkulila T, Vounatsou P, Kombe Y, Mayombana C, et al. Evolution of *Schistosoma haematobium*-related pathology over 24 months after treatment with praziquantel among school children in southeastern Tanzania. Am J Trop Med Hyg. 1998;59(5):775-81.

49. Hoekstra PT, Casacuberta-Partal M, van Lieshout L, Corstjens PLAM, Tsonaka R, Assare RK, et al. Efficacy of single versus four repeated doses of praziquantel against *Schistosoma mansoni* infection in school-aged children from Cote d'Ivoire based on Kato-Katz and POC-CCA: An open-label, randomised controlled trial (RePST). PLoS Negl Trop Dis. 2020;14(3).

50. Ibironke O, Koukounari A, Asaolu S, Moustaki I, Shiff C. Validation of a new test for *Schistosoma haematobium* based on detection of Dra1 DNA fragments in urine: evaluation through latent class analysis. PLoS Negl Trop Dis. 2012;6(1):e1464.

51. Inyang-Etoh PC, Ejezie GC, Useh MF, Inyang-Etoh EC. Efficacy of a combination of praziquantel and artesunate in the treatment of urinary schistosomiasis in Nigeria. Trans R Soc Trop Med Hyg. 2009;103(1):38-44.

52. Ismail MM, Attia MM, El-Badawy AA, Farghaly AM, Husein MH, Metwally A. Treatment of schistosomiasis with praziquantel among school children. J Egypt Soc Parasitol. 1994;24(3):487-94.

53. Ismail MM, Bruce JI, Attia M, Husein MH, Sabah AA, Tayel SE. The status of schistosomiasis before and after treatment among school pupils in a village in the Nile delta Egypt. J Egypt Soc Parasitol. 1988;18(1):287-96.

54. Jonge Nd, De Caluwe P, Hilberath GW, Krijger FW, Polderman AM, Deelder AM. Circulating anodic antigen levels in serum before and after chemotherapy with praziquantel in *Schistosomiasis mansoni*. Trans R Soc Trop Med Hyg. 1989;83(3):368-72.

55. Jonge Nd, Schommer G, Feldmeier H, Krijger FW, Dafalla AA, Bienzle U, et al. Mixed *Schistosoma haematobium* and *S. mansoni* infection: effect of different treatments on the serum level of circulating anodic antigen (CAA). Acta Trop. 1990;48(1):25-35.

56. Kabatereine NB, Kemijumbi J, Ouma JH, Sturrock RF, Butterworth AE, Madsen H, et al. Efficacy and side effects of praziquantel treatment in a highly endemic *Schistosoma mansoni* focus at Lake Albert, Uganda. Trans R Soc Trop Med Hyg. 2003;97(5):599-603.

57. Kabuyaya M, Chimbari MJ, Manyangadze T, Mukaratirwa S. Efficacy of praziquantel on *Schistosoma haematobium* and re-infection rates among school-going children in the Ndumo area of uMkhanyakude district, KwaZulu-Natal, South Africa. Infect Dis Poverty. 2017;6(1):83.

58. Kahama AI, Odek AE, Kihara RW, Vennervald BJ, Kombe Y, Nkulila T, et al. Urine circulating soluble egg antigen in relation to egg counts, hematuria, and urinary tract pathology before and after treatment in children infected with *Schistosoma haematobium* in Kenya. Am J Trop Med Hyg. 1999;61(2):215-9.

59. Kahama AI, Vennervald BJ, Kombe Y, Kihara RW, Ndzovu M, Mungai P, et al. Parameters associated with *Schistosoma haematobium* infection before and after chemotherapy in school children from two villages in the Coast province of Kenya. Trop Med Int Health. 1999;4(5):335-40.

60. Karanja DHS, Boyer AE, Strand M, Colley DG, Nahlen BL, Ouma JH, et al. Studies on schistosomiasis in western Kenya: II. Efficacy of praziquantel for treatment of schistosomiasis in persons coinfected with human immunodeficiency VIRUS-1. Am J Trop Med Hyg. 1998;59(2):307-11.

61. Katz N, Rocha RS, De Souza CP. Efficacy of alternating therapy with oxamniquine and praziquantel to treat *Schistosoma mansoni* in children following failure of first treatment. Am J Trop Med Hyg. 1991;44(5):509-12.

62. Keiser J, N'Guessan NA, Adoubryn KD, Silue KD, Vounatsou P, Hatz C, et al. Efficacy and safety of mefloquine, artesunate, mefloquine artesunate, and praziquantel against *Schistosoma haematobium*: randomized, exploratory open label trial. Clin Infect Dis. 2010;50(9):1205-13.

63. Keiser J, Silué KD, Adiossan LK, N'Guessan NA, Monsan N, Utzinger J, et al. Praziquantel, mefloquine-praziquantel, and mefloquine-artesunate-praziquantel against *Schistosoma haematobium*: A randomized, exploratory, open-label trial. PLoS Negl Trop Dis. 2014;8(7).

64. Kemal M, Tadesse G, Esmael A, Abay SM, Kebede T. *Schistosoma mansoni* infection among preschool age children attending Erer Health Center, Ethiopia and the response rate to praziquantel. BMC Res Notes. 2019;12(1):211-.

65. Khalil HM, El-Hawey AM, Baki MHA, El-Hadi HM, Sharara MOA. Evaluation of praziquantel efficiency in the treatment of schistosomiasis. J Egypt Soc Parasitol. 1986;16(2):403-12.

66. Kihara JH, Muhoho N, Njomo D, Mwobobia IK, Josyline K, Mitsui Y, et al. Drug efficacy of praziquantel and albendazole in school children in Mwea Division, Central Province, Kenya. Acta Trop. 2007;102(3):165-71.

67. Kihara JH, Njagi EN, Kenya EU, Mwanje MT, Odek AE, van Dam G, et al. Urinary soluble egg antigen levels in *Schistosoma haematobium* infection in relation to sex and age of Kenyan schoolchildren following praziquantel treatment. Trans R Soc Trop Med Hyg. 2009;103(10):1024-30.

68. Kiliku FM, Kimura E, Muhoho N, Migwi DK, Katsumata T. The usefulness of urinalysis reagent strips in selecting *Schistosoma haematobium* egg positives before and after treatment with praziquantel. J Trop Med Hyg. 1991;94(6):401-6.

69. Kimani BW, Mbugua AK, Kihara JH, Ng'ang'a M, Njomo DW. Safety, efficacy and acceptability of praziquantel in the treatment of *Schistosoma haematobium* in pre-school children of Kwale County, Kenya. PLoS Negl Trop Dis. 2018;12(10):e0006852.

70. Kimura E, Moji K, Uga S, Kiliku FM, Migwi DK, Mutua WR, et al. Effects of *Schistosoma haematobium* infection on mental test-scores of kenyan school-children. Trop Med Parasitol. 1992;43(3):155-8.

71. Kjetland EF, Mduluza T, Ndhlovu PD, Gomo E, Gwanzura L, Midzi N, et al. Genital schistosomiasis in women: a clinical 12-month in vivo study following treatment with praziquantel. Trans R Soc Trop Med Hyg. 2006;100(8):740-52.

72. Latham MC, Stephenson LS, Kurz KM, Kinoti SN. Metrifonate or praziquantel treatment improves physical fitness and appetite of Kenyan schoolboys with *Schistosoma haematobium* and hookworm infections. Am J Trop Med Hyg. 1990;43(2):170-9.

73. Massoud AAE, El Kholy AM, Anwar WA. Assessment on efficacy of praziquantel against *Schistosoma mansoni* infection. J Trop Med Hyg. 1984;87(3):119-21.

74. McMahon JE. Observations on praziquantel against *Schistosoma haematobium*. Arzneimittelforschung. 1981:579-80.

75. McMahon JE. A comparative trial of praziquantel, metrifonate and niridazole against *Schistosoma haematobium*. Ann Trop Med Parasit 1983;77(2):139-42.

76. McMahon JE, Kolstrup N. Praziquantel: a new schistosomicide against *Schistosoma haematobium*. Br Med J. 1979;2(6202):1396-9.

77. Mduluza T, Ndhlovu PD, Madziwa TM, Midzi N, Zinyama R, Turner CM, et al. The impact of repeated treatment with praziquantel of schistosomiasis in children under six years of age living in an endemic area for *Schistosoma haematobium* infection. Mem Inst Oswaldo Cruz. 2001;96 Suppl:157-64.

78. Metwally A, Bennett J, Botros S, Ebeid F, Elattar G. Impact of drug-dosage and brand on bioavailability and efficacy of praziquantel. Pharmacol Res. 1995;31(1):53-9.

79. Midzi N, Sangweme D, Zinyowera S, Mapingure MP, Brouwer KC, Kumar N, et al. Efficacy and side effects of praziquantel treatment against *Schistosoma haematobium* infection among primary school children in Zimbabwe. Trans R Soc Trop Med Hyg. 2008;102(8):759-66.

80. Mishra P, Colombe S, Paul N, Mlingi J, Tosiri I, Aristide C, et al. Insufficiency of annual praziquantel treatment to control *Schistosoma mansoni* infections in adult women: A longitudinal cohort study in rural Tanzania. PLoS Negl Trop Dis. 2019;13(11).

81. Mnkugwe RH, Minzi O, Kinung'hi S, Kamuhabwa A, Aklillu E. Efficacy and safety of praziquantel and dihydroartemisinin piperaquine combination for treatment and control of intestinal schistosomiasis: A randomized, non-inferiority clinical trial. PLoS Negl Trop Dis. 2020;14(9):e0008619-e.

82. Mohamed AA, Mahgoub HM, Magzoub M, Gasim GI, Eldein WN, Ahmed AeAA, et al. Artesunate plus sulfadoxine/pyrimethamine versus praziquantel in the treatment of *Schistosoma mansoni* in eastern Sudan. Trans R Soc Trop Med Hyg. 2009;103(10):1062-4.

83. Mohammed EH, Eltayeb M, Ibrahim H. Haematological and biochemical morbidity of *Schistosoma haematobium* in school children in Sudan. Sultan Qaboos Univ Med J. 2006;6(2):59-64.

84. Muhumuza S, Olsen A, Katahoire A, Kiragga AN, Nuwaha F. Effectiveness of a pre-treatment snack on the uptake of mass treatment for schistosomiasis in uganda: A cluster randomized trial. PLoS Med. 2014;11(5).

85. Munisi DZ, Buza J, Mpolya EA, Angelo T, Kinung'hi SM. The efficacy of single-dose versus double-dose praziquantel treatments on *Schistosoma mansoni* infections: Its implication on undernutrition and anaemia among primary schoolchildren in two on-shore communities, Northwestern Tanzania. Biomed Res Int. 2017;2017:7035025.

86. Muok EMO, Simiyu EW, Ochola EA, Ng'ang'a ZW, Secor WE, Karanja DMS, et al. Short report: Association between CD4(+) T-Lymphocyte counts and fecal excretion of *Schistosoma mansoni* eggs in patients coinfected with *S. mansoni* and human immunodeficiency virus before and after initiation of antiretroviral therapy. Am J Trop Med Hyg. 2013;89(1):42-5.

87. Mutapi F, Hagan P, Woolhouse MEJ, Mduluza T, Ndhlovu PD. Chemotherapy-induced, age-related changes in antischistosome antibody responses. Parasite Immunol. 2003;25(2):87-97.

88. Mutapi F, Ndhlovu PD, Hagan P, Spicer JT, Mduluza T, Turner CMR, et al. Chemotherapy accelerates the development of acquired immune responses to *Schistosoma haematobium* infection. J Infect Dis. 1998;178(1):289-93.

89. Mutapi F, Ndhlovu PD, Hagan P, Woolhouse MEJ. Changes in specific anti-egg antibody levels following treatment with praziquantel for *Schistosoma haematobium* infection in children. Parasite Immunol. 1998;20(12):595-600.

90. Mutapi F, Rujeni N, Bourke C, Mitchell K, Appleby L, Nausch N, et al. *Schistosoma haematobium* treatment in 1-5 year old children: Safety and efficacy of the antihelminthic drug praziquantel. PLoS Negl Trop Dis. 2011;5(5).

91. Mutsaka-Makuvaza MJ, Matsena-Zingoni Z, Tshuma C, Ray S, Zhou XN, Webster B, et al. Reinfection of urogenital schistosomiasis in pre-school children in a highly endemic district in Northern Zimbabwe: A 12 months compliance study 11 medical and health sciences 1117 public health and health services. Infect Dis Poverty. 2018;7(1).

92. Mwanakasale V, Siziya S, Mwansa J, Koukounari A, Fenwick A. Impact of iron supplementation on schistosomiasis control in Zambian school children in a highly endemic area. Malawi Med J. 2009;21(1):12-8.

93. N'Goran EK, Utzinger J, N'Guessan AN, Muller I, Zamble K, Lohourignon KL, et al. Reinfection with *Schistosoma haematobium* following school-based chemotherapy with praziquantel in four highly endemic villages in Cote d'Ivoire. Trop Med Int Health. 2001;6(10):817-25.

94. Nalugwa A, Nuwaha F, Tukahebwa EM, Olsen A. Single versus double dose praziquantel comparison on efficacy and *Schistosoma mansoni* re-infection in preschool-age children in Uganda: A randomized controlled trial. PLoS Negl Trop Dis. 2015;9(5):e0003796.

95. Navaratnam AMD, Sousa-Figueiredo JC, Stothard JR, Kabatereine NB, Fenwick A, Mutumba-Nakalembe MJ. Efficacy of praziquantel syrup versus crushed praziquantel tablets in the treatment of intestinal schistosomiasis in Ugandan preschool children, with observation on compliance and safety. Trans R Soc Trop Med Hyg. 2012;106(7):400-7.

96. Nega B, Gundersen SG, Fekadu A, Hailu B, Girmay M, Teferi G. Praziquantel side effects and efficacy related to *Schistosoma mansoni* egg loads and morbidity in primary school children in north-east Ethiopia. Acta Trop. 1999;72(1):53-63.

97. Nkengazong L, Njiokou F, Teukeng F, Enyong P, Wanji S. Reassessment of endemicity level of urinary schistosomiasis in the Kotto-Barombi focus (South West Cameroon) and impact of mass drug administration (MDA) on the parasitic indices. J Cell Anim Biol. 2009;3(9):159-64.

98. Obonyo CO, Muok EMO, Mwinzi PNM. Efficacy of artesunate with sulfalene plus pyrimethamine versus praziquantel for treatment of *Schistosoma mansoni* in Kenyan children: an open-label randomised controlled trial. Lancet Infect Dis. 2010;10(9):603-11.

99. Ofoezie IE. Patterns of reinfection following praziquantel treatment of urinary schistosomiasis at a period of low transmission. Acta Trop. 2000;75(1):123-6.

100. Olds GR, King C, Hewlett J, Olveda R, Wu G, Ouma J, et al. Double-blind placebo-controlled study of concurrent administration of albendazole and praziquantel in schoolchildren with schistosomiasis and geohelminths. J Infect Dis. 1999;179(4):996-1003.

101. Olliaro PL, Vaillant MT, Belizario VJ, Lwambo NJS, Ouldabdallahi M, Pieri OS, et al. A multicentre randomized controlled trial of the efficacy and safety of single-dose praziquantel at 40 mg/kg vs. 60 mg/kg for treating intestinal schistosomiasis in the Philippines, Mauritania, Tanzania and Brazil. PLoS Negl Trop Dis. 2011;5(6).

102. Olsen A, Nawiri J, Friis H. The impact of iron supplementation on reinfection with intestinal helminths and *Schistosoma mansoni* in western Kenya. Trans R Soc Trop Med Hyg. 2000;94(5):493-9.

103. Olsen A, Thiong'o FW, Ouma JH, Mwaniki D, Magnussen P, Michaelsen KF, et al. Effects of multimicronutrient supplementation on helminth reinfection: a randomized, controlled trial in Kenyan schoolchildren. Trans R Soc Trop Med Hyg. 2003;97(1):109-14.

104. Opara KN, Mbagwu HC, Ekpo UF. Urinary schistosomiasis in two endemic communities of Cross River Basin: prevalence, intensity, and treatment. MSJM. 2003;3(1):28-34.

105. Osakunor DNM, Mduluza T, Midzi N, Chase-Topping M, Mutsaka-Makuvaza MJ, Chimponda T, et al. Dynamics of paediatric urogenital schistosome infection, morbidity and treatment: a longitudinal study among preschool children in Zimbabwe. BMJ Glob Health. 2018;3(2):e000661.

106. Ouldabdallahi M, Ousmane B, Ouldbezeid M, Mamadou D, Konate L, Chitsulo L. Comparison of the efficacy and safety of praziquantel administered in single dose of 40 versus 60 mg/kg for treating urinary schistosomiasis in Mauritania. Bull Soc Pathol Exot. 2013;106(3):167-9.

107. Oyediran AB, Kofie BA, Bammeke AO, Bamgboye EA. Clinical experience with praziquantel in the treatment of Nigerian patients infected with *S. haematobium*. Arzneimittelforschung. 1981;31(3a):581-4.

108. Polderman AM, Gryseels B, De Caluwe P. Cure rates and egg reduction in treatment of intestinal schistosomiasis with oxamniquine and praziquantel in Maniema, Zaire. Trans R Soc Trop Med Hyg. 1988;82(1):115-6.

109. Reimert CM, Mshinda HM, Hatz CF, Kombe Y, Nkulila T, Poulsen LK, et al. Quantitative assessment of eosinophiluria in *Schistosoma haematobium* infections: A new marker of infection and bladder morbidity. Am J Trop Med Hyg. 2000;62(1):19-28.

110. Reimert CM, Ouma JH, Mwanje MT, Magak P, Poulsen LK, Vennervald BJ, et al. Indirect assessment of eosinophiluria in urinary schistosomiasis using eosinophil Cationic Protein (ECP) and Eosinophil Protein-X (EPX). Acta Trop. 1993;54(1):1-12.

111. Reta B, Erko B. Efficacy and side effects of praziquantel in the treatment for *Schistosoma mansoni* infection in school children in Senbete Town, Northeastern Ethiopia. Trop Med Int Health. 2013;18(11):1338-43.

112. Roberts M, Butterworth AE, Kimani G, Kamau T, Fulford AJC, Dunne DW, et al. Immunity after treatment of human schistosomiasis - association between cellular-responses and resistance to reinfection. Infection and Immunity. 1993;61(12):4984-93.

113. Rugemalila JB, Asila J, Chimbe A. Randomized comparative trials of single doses of the newer antischistosomal drugs at Mwanza, Tanzania .1. Praziquantel and oxamniquine for the treatment of *Schistosomiasis mansoni*. J Trop Med Hyg. 1984;87(6):231-5.

114. Saathoff E, Olsen A, Magnussen P, Kvalsvig JD, Becker W, Appleton CC. Patterns of *Schistosoma haematobium* infection, impact of praziquantel treatment and re-infection after treatment in a cohort of schoolchildren from rural KwaZulu-Natal/South Africa. BMC Infect Dis. 2004;4.

115. Sacko M, Magnussen P, Traore M, Landoure A, Doucoure A, Reimert CM, et al. The effect of single dose versus two doses of praziquantel on *Schistosoma haematobium* infection and pathology among school-aged children in Mali. Parasitology. 2009;136(13):1851-7.

116. Satti MZ, Lind P, Vennervald BJ, Sulaiman SM, Daffalla AA, Ghalib HW. Specific immunoglobulin measurements related to exposure and resistance to *Schistosoma mansoni* infection in Sudanese canal cleaners. Clin Exp Immunol. 1996;106(1):45-54.

117. Scherrer AU, Sjoeberg MK, Allangba A, Traore M, Lohourignon LK, Tschannen AB, et al. Sequential analysis of helminth egg output in human stool samples following albendazole and praziquantel administration. Acta Trop. 2009;109(3):226-31.

118. Schutte CH, Osman Y, Van Deventer JM, Mosese G. Effectiveness of praziquantel against the South African strains of *Schistosoma haematobium* and *S. mansoni*. S Afr Med J. 1983;64(1):7-10.

119. Scott JT, Turner CMR, Mutapi F, Woolhouse MEJ, Ndhlovu PD, Hagan P. Cytokine responses to mitogen and *Schistosoma haematobium* antigens are different in children with distinct infection histories. Parasite Immunol. 2001;23(10):519-26.

120. Senghor B, Diaw OT, Doucoure S, Seye M, Diallo A, Talla I, et al. Impact of annual praziquantel treatment on urogenital schistosomiasis in a seasonal transmission focus in Central Senegal. PLoS Negl Trop Dis. 2016;10(3):e0004557.

121. Senghor B, Diaw OT, Doucoure S, Sylla SN, Seye M, Talla I, et al. Efficacy of praziquantel against urinary schistosomiasis and reinfection in Senegalese school children where there is a single well-defined transmission period. Parasit Vectors. 2015;8:362.

122. Simonsen PE, Nega A, Furu P. Intestinal schistosomiasis among children in a labour village of Wonji Sugar Estate, Ethiopia. East Afr Med J. 1990;67(8):532-8.

123. Sissoko MS, Dabo A, Traore H, Diallo M, Traore B, Konate D, et al. Efficacy of artesunate plus sulfamethoxypyrazine/pyrimethamine versus praziquantel in the treatment of *Schistosoma haematobium* in children. PLoS One. 2009;4(10).

124. Snyman JR, Sommers de K. Effect of levamisole on the immune response of patients with schistosomiasis after treatment with praziquantel. Clin Drug Investig. 1998;15(6):483-9.

125. Snyman JR, Sommers KD, Steinmann MA, Lizamore DJ. Effects of calcitriol on eosinophil activity and antibody responses in patients with schistosomiasis. Eur J Clin Pharmacol 1997;52(4):277-80.

126. Sousa-Figueiredo JC, Betson M, Atuhaire A, Arinaitwe M, Navaratnam AM, Kabatereine NB, et al. Performance and safety of praziquantel for treatment of intestinal schistosomiasis in infants and preschool children. PLoS Negl Trop Dis. 2012;6(10):e1864.

127. Sousa-Figueiredo JC, Pleasant J, Day M, Betson M, Rollinson D, Montresor A, et al. Treatment of intestinal schistosomiasis in Ugandan preschool children: best diagnosis, treatment efficacy and side-effects, and an extended praziquantel dosing pole. Int Health. 2010;2(2):103-13.

128. Stete K, Krauth SJ, Coulibaly JT, Knopp S, Hattendorf J, Muller I, et al. Dynamics of *Schistosoma haematobium* egg output and associated infection parameters following treatment with praziquantel in school-aged children. Parasit Vectors. 2012;5(298).

129. Taddese K, Zein ZA. Comparison between the efficacy of oxamniquine and praziquantel in the treatment of *Schistosoma mansoni* infections on a sugar estate in Ethiopia. Ann Trop Med Parasit 1988;82(2):175-80.

130. Tchuente LA, Shaw DJ, Polla L, Cioli D, Vercruysse J. Efficacy of praziquantel against *Schistosoma haematobium* infection in children. Am J Trop Med Hyg. 2004;71(6):778-82.

131. Tesfie A, Getnet G, Abere A, Yihenew G, Belete Y, Kassa M, et al. Praziquantel is an effective drug for the treatment of *Schistosoma mansoni* infection among school-aged children in Northwest Ethiopia. Trop Med Int Health. 2020;48(1).

132. Tetteh-Quarcoo PB, Forson PO, Amponsah SK, Ahenkorah J, Opintan JA, Ocloo JEY, et al. Persistent urogenital schistosomiasis and its associated morbidity in endemic communities within Southern Ghana: Suspected praziquantel resistance or reinfection? Med Sci (Basel). 2020;8(1).

133. Thiong'o FW, Mbugua GG, Ouma JH, Sturrock RK. Efficacy of oxamniquine and praziquantel in school children from two *Schistosoma mansoni* endemic areas. East Afr Med J. 2002;79(1):29-33.

134. Tweyongyere R, Mawa PA, Emojong NO, Mpairwe H, Jones FM, Duong T, et al. Effect of praziquantel treatment of *Schistosoma mansoni* during pregnancy on intensity of infection and antibody responses to schistosome antigens: results of a randomised, placebo-controlled trial. BMC Infect Dis. 2009;9.

135. Utzinger J, Booth M, N'Goran EK, Muller I, Tanner M, Lengeler C. Relative contribution of day-to-day and intra-specimen variation in faecal egg counts of *Schistosoma mansoni* before and after treatment with praziquantel. Parasitology. 2001;122:537-44.

136. Utzinger J, N'Goran EK, N'Dri A, Lengeler C, Tanner M. Efficacy of praziquantel against *Schistosoma mansoni* with particular consideration for intensity of infection. Trop Med Int Health. 2000;5(11):771-8.

137. Wami WM, Nausch N, Midzi N, Gwisai R, Mduluza T, Woolhouse ME, et al. Comparative assessment of health benefits of praziquantel treatment of urogenital schistosomiasis in preschool and primary school-aged children. Biomed Res Int. 2016;2016:9162631.

138. Webster BL, Diaw OT, Seye MM, Faye DS, Stothard JR, Sousa-Figueiredo JC, et al. Praziquantel treatment of school children from single and mixed infection foci of intestinal and urogenital schistosomiasis along the Senegal River Basin: monitoring treatment success and re-infection patterns. Acta Trop. 2013;128(2):292-302.

139. Webster M, Fallon PG, Fulford AJC, Butterworth AE, Ouma JH, Kimani G, et al. Effect of praziquantel and oxamniquine treatment on human isotype responses to *Schistosoma mansoni*: Elevated IgE to adult worm. Parasite Immunol. 1997;19(7):333-5.

140. Wilkins HA, Blumenthal UJ, Hagan P, Hayes RJ, Tulloch S. Resistance to reinfection after treatment of urinary schistosomiasis. Trans R Soc Trop Med Hyg. 1987;81(1):29-35.

141. Wilkins HA, Moore PJ. Comparative trials of regimes for the treatment of urinary schistosomiasis in The Gambia. J Trop Med Hyg. 1987;90(2):83-92.

142. Wilson S, Jones FM, Kenty LC, Mwatha JK, Kimani G, Kariuki HC, et al. Posttreatment changes in cytokines induced by *Schistosoma mansoni* egg and worm antigens: dissociation of immunity- and morbidity-associated type 2 responses. J Infect Dis. 2014;209(11):1792-800.

143. Woldegerima E, Bayih AG, Tegegne Y, Aemero M, Zeleke AJ. Prevalence and reinfection rates of *Schistosoma mansoni* and praziquantel efficacy against the parasite among primary school children in Sanja town, Northwest Ethiopia. J Parasitol Res. 2019;2019 (no pagination).

144. Wu WX. Clinical observation on the treatment of 1627 cases of schistosomiasis haematobia with praziquantel of different dosages. Chin J Parasitol Parasit Dis. 1994;12(4):288-90.

145. Yimam Y, Degarege A, Erko B. Effect of anthelminthic treatment on helminth infection and related anaemia among school-age children in northwestern Ethiopia. BMC Infect Dis. 2016;16(1).

146. Zinyowera S, Muchaneta-Kubara CE, Mutapi F, Midzi N, Ndlovu PD, Mduluza T. Changes in the humoral immune responses after chemotherapy in single and co-infected individuals with *Schisosoma haematobium* and Plasmodium falciparum. Cent Afr J Med. 2006;52(9-12):104-11.
