# Supplementary material for: Efficacy of praziquantel has been maintained over four decades (from 1977 to 2018): A systematic review and meta-analysis of factors influence its efficacy
Source: PLoS Negl Trop Dis. 2021 Mar 17;15(3):e0009189. doi: 10.1371/journal.pntd.0009189 (PMC7968639; doi:10.1371/journal.pntd.0009189)
Supplement: S2 Appendix — When a study met and/or reported each criterion a score of one was given. A study categorized “high quality” when a total score was 6–7, “medium” with 3–5, and “low” with 0–2. (DOCX) [file pntd.0009189.s002.docx]

**Appendix 2. Quality assurance of studies. When a study met and/or reported each criterion a score of one was given. A study categorized “high quality” when a total score was 6-7, “medium” with 3-5, and “low” with 0-2.**

| Article (first author and publication year) | Population | Treatment year | Description of laboratory diagnostic methods | Pretreatment infection intensity | Multiple samples on different days | Multiple tests of a sample | Type of mean to calculate CR and/or ERR | Total score (out of 7) | Quality |
| --- | --- | --- | --- | --- | --- | --- | --- | --- | --- |
| Abu-Elyazeed 1993 | 1 | 1 | 1 | 0 | 1 | 1 | 0 | 5 | Medium |
| Abu-Elyazeed 1998 | 1 | 1 | 1 | 0 | 0 | 0 | 0 | 3 | Medium |
| Adoubryn 2012 | 1 | 1 | 1 | 0 | 0 | 0 | 0 | 3 | Medium |
| Alharbi 2019 | 1 | 1 | 1 | 0 | 0 | 0 | 0 | 3 | Medium |
| Anwar 1993 | 1 | 0 | 1 | 1 | 1 | 1 | 1 | 6 | High |
| Augusto 2009 | 1 | 1 | 1 | 1 | 1 | 1 | 1 | 7 | High |
| Bajiro 2016 | 1 | 1 | 0 | 0 | 0 | 0 | 1 | 3 | Medium |
| Banwat 2011 | 1 | 1 | 1 | 0 | 0 | 0 | 0 | 3 | Medium |
| Barakat 2011 | 1 | 0 | 1 | 0 | 0 | 1 | 1 | 4 | Medium |
| Barda 2016 | 1 | 1 | 1 | 1 | 0 | 1 | 1 | 6 | High |
| Ben 2017 | 1 | 1 | 1 | 1 | 0 | 0 | 0 | 4 | Medium |
| Black 2009 | 1 | 1 | 1 | 0 | 1 | 1 | 0 | 5 | Medium |
| Borrmann 2001 | 1 | 1 | 1 | 1 | 1 | 1 | 1 | 7 | High |
| Botros 2005 | 1 | 0 | 1 | 1 | 0 | 1 | 1 | 5 | Medium |
| Burchard 1984 | 1 | 0 | 0 | 0 | 0 | 0 | 0 | 1 | Low |
| Bustinduy 2013 | 1 | 1 | 1 | 0 | 1 | 1 | 0 | 5 | Medium |
| Bustinduy 2016 | 1 | 1 | 1 | 1 | 1 | 1 | 1 | 7 | High |
| Butterworth 1991 | 1 | 1 | 1 | 1 | 0 | 1 | 1 | 6 | High |
| Cabello 2016 | 1 | 1 | 1 | 1 | 0 | 1 | 1 | 6 | High |
| Campagne 2001 | 1 | 1 | 1 | 1 | 0 | 0 | 1 | 5 | Medium |
| Chisango 2019 | 1 | 1 | 1 | 0 | 1 | 1 | 1 | 6 | High |
| Chuks 1987 | 1 | 0 | 1 | 1 | 0 | 0 | 1 | 4 | Medium |
| Clercq 2000 | 1 | 1 | 1 | 1 | 0 | 0 | 1 | 5 | Medium |
| Clercq 2002 | 1 | 1 | 1 | 1 | 0 | 0 | 1 | 5 | Medium |
| Coulibaly 2017 | 1 | 1 | 1 | 1 | 0 | 1 | 1 | 6 | High |
| Coulibaly 2018 | 1 | 1 | 1 | 1 | 1 | 1 | 1 | 7 | High |
| Davis 1981 | 1 | 0 | 1 | 0 | 1 | 1 | 0 | 4 | Medium |
| Degu 2002 | 1 | 1 | 1 | 0 | 0 | 0 | 0 | 3 | Medium |
| Doehring 1986 | 1 | 0 | 1 | 1 | 1 | 1 | 1 | 6 | High |
| El-Ghandour 1989 | 1 | 0 | 1 | 1 | 0 | 0 | 0 | 3 | Medium |
| El-Hawey 1992 | 1 | 0 | 0 | 1 | 0 | 0 | 1 | 3 | Medium |
| El-Morshedy 1996 | 1 | 0 | 1 | 1 | 0 | 0 | 1 | 4 | Medium |
| Elbasheir 2020 | 1 | 1 | 1 | 0 | 0 | 1 | 0 | 4 | Medium |
| Erko 2012 | 1 | 1 | 1 | 1 | 0 | 0 | 1 | 5 | Medium |
| Farid 1984 | 0 | 0 | 1 | 1 | 0 | 0 | 1 | 3 | Medium |
| Friis 1989 | 1 | 0 | 1 | 1 | 0 | 0 | 1 | 4 | Medium |
| Garba 2001 | 1 | 1 | 1 | 1 | 1 | 1 | 1 | 7 | High |
| Grogan 1996 | 1 | 0 | 1 | 1 | 0 | 1 | 1 | 5 | Medium |
| Groning 1985 | 0 | 0 | 0 | 0 | 0 | 0 | 0 | 0 | Low |
| Gryseels 1987 | 1 | 1 | 1 | 1 | 0 | 1 | 1 | 6 | High |
| Gryseels 1991 | 1 | 1 | 1 | 0 | 0 | 1 | 0 | 4 | Medium |
| Guidi 2010 | 1 | 1 | 1 | 0 | 0 | 1 | 0 | 4 | Medium |
| Guisse 1997 | 1 | 1 | 1 | 0 | 1 | 1 | 1 | 6 | High |
| Gyoten 1992 | 1 | 1 | 1 | 1 | 1 | 1 | 1 | 7 | High |
| Hailu 2018 | 1 | 1 | 1 | 0 | 0 | 0 | 0 | 3 | Medium |
| Hamm 2009 | 1 | 0 | 0 | 1 | 0 | 0 | 1 | 3 | Medium |
| Hassanein 1997 | 1 | 0 | 1 | 1 | 1 | 1 | 1 | 6 | High |
| Hatz 1998 | 1 | 1 | 1 | 0 | 1 | 1 | 0 | 5 | Medium |
| Hoekstra 2020 | 1 | 0 | 1 | 1 | 0 | 1 | 0 | 4 | Medium |
| Ibironke 2012 | 1 | 1 | 1 | 0 | 0 | 0 | 0 | 3 | Medium |
| Inyang-Etoh 2009 | 1 | 1 | 1 | 1 | 1 | 1 | 1 | 7 | High |
| Ismail 1988 | 1 | 0 | 1 | 0 | 0 | 0 | 0 | 2 | Low |
| Ismail 1994 | 1 | 0 | 1 | 0 | 0 | 0 | 1 | 3 | Medium |
| Jonge 1989 | 1 | 0 | 1 | 1 | 1 | 1 | 1 | 6 | High |
| Jonge 1990 | 1 | 0 | 1 | 1 | 1 | 1 | 1 | 6 | High |
| Kabatereine 2003 | 1 | 1 | 1 | 0 | 1 | 1 | 0 | 5 | Medium |
| Kabuyaya 2017 | 1 | 1 | 1 | 1 | 0 | 0 | 1 | 5 | Medium |
| Kahama 1999 | 1 | 0 | 1 | 1 | 1 | 1 | 1 | 6 | High |
| Kahama 1999 | 1 | 0 | 1 | 0 | 1 | 1 | 0 | 4 | Medium |
| Karanja 1998 | 1 | 0 | 1 | 0 | 0 | 1 | 0 | 3 | Medium |
| Katz 1991 | 1 | 0 | 1 | 0 | 0 | 1 | 0 | 3 | Medium |
| Keiser 2010 | 1 | 1 | 1 | 1 | 0 | 0 | 1 | 5 | Medium |
| Keiser 2014 | 1 | 1 | 1 | 1 | 0 | 0 | 1 | 5 | Medium |
| Kemal 2019 | 1 | 0 | 1 | 1 | 1 | 1 | 0 | 5 | Medium |
| Khalil 1986 | 0 | 0 | 1 | 0 | 0 | 0 | 0 | 1 | Low |
| Kihara 2007 | 1 | 1 | 1 | 1 | 0 | 0 | 1 | 5 | Medium |
| Kihara 2009 | 1 | 1 | 1 | 1 | 0 | 1 | 1 | 6 | High |
| Kiliku 1991 | 1 | 1 | 0 | 1 | 0 | 0 | 1 | 4 | Medium |
| Kimani 2018 | 1 | 1 | 1 | 1 | 0 | 1 | 1 | 6 | High |
| Kimura 1992 | 1 | 0 | 1 | 1 | 0 | 0 | 1 | 4 | Medium |
| Kjetland 2006 | 1 | 0 | 1 | 0 | 1 | 1 | 0 | 4 | Medium |
| Latham 1990 | 1 | 0 | 1 | 1 | 0 | 0 | 1 | 4 | Medium |
| Massoud 1984 | 1 | 0 | 0 | 0 | 0 | 0 | 1 | 2 | Low |
| McMahon 1979 | 0 | 0 | 1 | 1 | 1 | 1 | 1 | 5 | Medium |
| McMahon 1981 | 0 | 0 | 1 | 0 | 1 | 1 | 0 | 3 | Medium |
| McMahon 1983 | 0 | 0 | 1 | 1 | 1 | 1 | 1 | 5 | Medium |
| Mduluza 2001 | 1 | 0 | 1 | 1 | 1 | 1 | 1 | 6 | High |
| Metwally 1995 | 1 | 0 | 1 | 1 | 1 | 1 | 1 | 6 | High |
| Midzi 2008 | 1 | 1 | 1 | 1 | 0 | 0 | 1 | 5 | Medium |
| Mishra 2019 | 1 | 0 | 1 | 1 | 0 | 1 | 0 | 4 | Medium |
| Mnkugwe 2020 | 1 | 0 | 1 | 1 | 1 | 1 | 0 | 5 | Medium |
| Mohamed 2009 | 1 | 1 | 1 | 0 | 1 | 1 | 0 | 5 | Medium |
| Mohammed 2006 | 1 | 0 | 1 | 0 | 0 | 0 | 0 | 2 | Low |
| Muhumuza 2014 | 1 | 1 | 1 | 1 | 1 | 1 | 1 | 7 | High |
| Munisi 2017 | 1 | 1 | 1 | 1 | 0 | 1 | 1 | 6 | High |
| Muok 2013 | 1 | 0 | 1 | 0 | 0 | 1 | 0 | 3 | Medium |
| Mutapi 1998 | 1 | 1 | 1 | 1 | 1 | 1 | 1 | 7 | High |
| Mutapi 1998 | 1 | 1 | 1 | 1 | 1 | 1 | 1 | 7 | High |
| Mutapi 2003 | 1 | 0 | 1 | 1 | 1 | 1 | 1 | 6 | High |
| Mutapi 2011 | 1 | 0 | 1 | 1 | 1 | 1 | 1 | 6 | High |
| Mutsaka-Makuvaza 2018 | 1 | 1 | 1 | 1 | 1 | 1 | 1 | 7 | High |
| Mwanakasale 2009 | 1 | 1 | 1 | 1 | 1 | 1 | 1 | 7 | High |
| N'Goran 2001 | 1 | 1 | 0 | 0 | 1 | 1 | 0 | 4 | Medium |
| Nalugwa 2015 | 1 | 1 | 1 | 1 | 1 | 1 | 1 | 7 | High |
| Navaratnam 2012 | 1 | 1 | 1 | 1 | 0 | 1 | 1 | 6 | High |
| Nega 1999 | 1 | 1 | 1 | 1 | 0 | 0 | 1 | 5 | Medium |
| Nkengazong 2009 | 1 | 1 | 1 | 1 | 0 | 0 | 1 | 5 | Medium |
| Obonyo 2010 | 1 | 1 | 1 | 1 | 0 | 0 | 1 | 5 | Medium |
| Ofoezie 2000 | 1 | 1 | 0 | 1 | 0 | 0 | 1 | 4 | Medium |
| Olds 1999 | 1 | 0 | 1 | 0 | 1 | 1 | 1 | 5 | Medium |
| Olliaro 2011 | 1 | 1 | 1 | 1 | 1 | 1 | 1 | 7 | High |
| Olsen 2000 | 1 | 1 | 1 | 0 | 1 | 1 | 0 | 5 | Medium |
| Olsen 2003 | 1 | 1 | 1 | 1 | 1 | 1 | 1 | 7 | High |
| Opara 2003 | 1 | 0 | 1 | 1 | 0 | 0 | 0 | 3 | Medium |
| Osakunor 2018 | 1 | 1 | 1 | 0 | 1 | 1 | 1 | 6 | High |
| Ouldabdallahi 2013 | 1 | 1 | 1 | 0 | 0 | 0 | 0 | 3 | Medium |
| Oyediran 1981 | 1 | 0 | 0 | 0 | 0 | 0 | 1 | 2 | Low |
| Polderman 1988 | 1 | 0 | 1 | 0 | 1 | 1 | 0 | 4 | Medium |
| Reimert 1993 | 1 | 0 | 1 | 0 | 0 | 0 | 0 | 2 | Low |
| Reimert 2000 | 1 | 0 | 1 | 1 | 1 | 1 | 1 | 6 | High |
| Reta 2013 | 1 | 1 | 1 | 1 | 0 | 0 | 1 | 5 | Medium |
| Roberts 1993 | 1 | 1 | 1 | 1 | 1 | 1 | 1 | 7 | High |
| Rugemalila 1984 | 1 | 0 | 0 | 0 | 0 | 0 | 1 | 2 | Low |
| Saathoff 2004 | 1 | 1 | 1 | 1 | 0 | 0 | 0 | 4 | Medium |
| Sacko 2009 | 1 | 0 | 1 | 0 | 1 | 1 | 0 | 4 | Medium |
| Satti 1996 | 1 | 0 | 1 | 1 | 1 | 1 | 1 | 6 | High |
| Scherrer 2009 | 1 | 1 | 1 | 1 | 0 | 0 | 1 | 5 | Medium |
| Schutte 1983 | 1 | 0 | 1 | 1 | 1 | 1 | 1 | 6 | High |
| Scott 2001 | 1 | 1 | 1 | 0 | 1 | 1 | 0 | 5 | Medium |
| Senghor 2015 | 1 | 1 | 1 | 1 | 0 | 0 | 1 | 5 | Medium |
| Senghor 2016 | 1 | 1 | 1 | 0 | 0 | 0 | 0 | 3 | Medium |
| Simonsen 1990 | 1 | 1 | 1 | 1 | 0 | 1 | 1 | 6 | High |
| Sissoko 2009 | 1 | 1 | 1 | 1 | 1 | 1 | 1 | 7 | High |
| Snyman 1997 | 1 | 0 | 1 | 1 | 0 | 0 | 1 | 4 | Medium |
| Snyman 1998 | 1 | 0 | 1 | 1 | 1 | 1 | 1 | 6 | High |
| Sousa-Figueiredo 2010 | 1 | 1 | 1 | 1 | 1 | 1 | 1 | 7 | High |
| Sousa-Figueiredo 2012 | 1 | 0 | 1 | 1 | 1 | 1 | 1 | 6 | High |
| Stete 2012 | 1 | 1 | 1 | 1 | 1 | 1 | 1 | 7 | High |
| Taddese 1988 | 1 | 1 | 1 | 1 | 0 | 1 | 1 | 6 | High |
| Tchuente 2004 | 1 | 1 | 1 | 1 | 0 | 1 | 1 | 6 | High |
| Tesfie 2020 | 1 | 0 | 1 | 1 | 0 | 0 | 0 | 3 | Medium |
| Tetteh-Quarcoo 2020 | 1 | 0 | 1 | 1 | 0 | 0 | 0 | 3 | Medium |
| Thiong'o 2002 | 1 | 1 | 1 | 1 | 1 | 1 | 1 | 7 | High |
| Tweyongyere 2009 | 1 | 1 | 1 | 0 | 0 | 0 | 0 | 3 | Medium |
| Utzinger 2000 | 1 | 1 | 1 | 1 | 1 | 1 | 1 | 7 | High |
| Utzinger 2001 | 1 | 1 | 1 | 1 | 1 | 1 | 1 | 7 | High |
| Wami 2016 | 1 | 1 | 1 | 1 | 1 | 1 | 1 | 7 | High |
| Webster 1997 | 1 | 0 | 0 | 1 | 0 | 0 | 1 | 3 | Medium |
| Webster 2013 | 1 | 1 | 1 | 1 | 1 | 1 | 1 | 7 | High |
| Wilkins 1987 | 1 | 0 | 1 | 1 | 1 | 1 | 1 | 6 | High |
| Wilkins 1987 | 1 | 1 | 1 | 1 | 1 | 1 | 1 | 7 | High |
| Wilson 2014 | 1 | 0 | 1 | 1 | 1 | 1 | 1 | 6 | High |
| Woldegerima 2019 | 1 | 0 | 1 | 1 | 0 | 1 | 0 | 4 | Medium |
| Wu 1994 | 1 | 0 | 0 | 0 | 0 | 0 | 0 | 1 | Low |
| Yimam 2016 | 1 | 1 | 1 | 1 | 0 | 0 | 1 | 5 | Medium |
| Zinyowera 2006 | 1 | 0 | 0 | 1 | 1 | 1 | 1 | 5 | Medium |
